# Virulence factor discovery identifies associations between the Fic gene family and Fap2^+^ fusobacteria in colorectal cancer microbiomes

**DOI:** 10.1128/mbio.03732-24

**Published:** 2025-01-14

**Authors:** Geicho Nakatsu, Duhyun Ko, Monia Michaud, Eric A. Franzosa, Xochitl C. Morgan, Curtis Huttenhower, Wendy S. Garrett

**Affiliations:** 1 Department of Immunology and Infectious Diseases, Harvard T.H. Chan School of Public Health, Boston, Massachusetts, USA; 2 Harvard T.H. Chan Microbiome in Public Health Center, Boston, Massachusetts, USA; 3 Department of Biostatistics, Harvard T.H. Chan School of Public Health, Boston, Massachusetts, USA; 4 Broad Institute of MIT and Harvard, Cambridge, Massachusetts, USA; 5 Department of Medical Oncology, Dana-Farber Cancer Institute, Boston, Massachusetts, USA; Rutgers The State University of New Jersey, Piscataway, New Jersey, USA

**Keywords:** *Fusobacterium animalis*, virulence factor, fap2, Fic, oncomicrobe

## Abstract

*Fusobacterium* is a bacterium associated with colorectal cancer (CRC) tumorigenesis, progression, and metastasis. Fap2 is a fusobacteria-specific outer membrane galactose-binding lectin that mediates *Fusobacterium* adherence to and invasion of CRC tumors. Advances in omics analyses provide an opportunity to profile and identify microbial genomic features that correlate with the cancer-associated bacterial virulence factor Fap2. Here, we analyze genomes of *Fusobacterium* colon tumor isolates and find that a family of post-translational modification enzymes containing Fic domains is associated with Fap2 positivity in these strains. We demonstrate that Fic family genes expand with the presence of Fap2 in the fusobacterial pangenome. Through comparative genomic analysis, we find that Fap2^+^ Fusobacteriota are highly enriched with Fic gene families compared to other cancer-associated and human gut microbiome bacterial taxa. Using a global data set of CRC shotgun metagenomes, we show that fusobacterial Fic and Fap2 genes frequently co-occur in the fecal microbiomes of individuals with late-stage CRC. We further characterize specific Fic gene families harbored by Fap2^+^ *Fusobacterium animalis* genomes and detect recombination events and elements of horizontal gene transfer via synteny analysis of Fic gene loci. Exposure of a *F. animalis* strain to a colon adenocarcinoma cell line increases gene expression of fusobacterial Fic and virulence-associated adhesins. Finally, we demonstrate that Fic proteins are synthesized by *F. animalis* as Fic peptides are detectable in *F. animalis* monoculture supernatants. Taken together, our study uncovers Fic genes as potential virulence factors in Fap2^+^ fusobacterial genomes.

**IMPORTANCE:**

Accumulating data support that bacterial members of the intra-tumoral microbiota critically influence colorectal cancer progression. Yet, relatively little is known about non-adhesin fusobacterial virulence factors that may influence carcinogenesis. Our genomic analysis and expression assays in fusobacteria identify Fic domain-containing genes, well-studied virulence factors in pathogenic bacteria, as potential fusobacterial virulence features. The Fic family proteins that we find are encoded by fusobacteria and expressed by *Fusobacterium animalis* merit future investigation to assess their roles in colorectal cancer development and progression.

## INTRODUCTION

*Fusobacterium* is associated with colorectal cancer (CRC), the third leading cause of cancer-related death worldwide (1–5). Numerous studies have linked the presence of intra-tumoral *Fusobacterium* with CRC tumorigenesis (6), metastasis (7), and therapeutic and preventative strategies (8–10). The majority of studies that investigate the molecular factors underlying fusobacterial virulence mechanisms have focused on its adhesins, principally Fap2 and FadA (11–16).

Researching the virulence factors of *Fusobacterium* is an ongoing effort with the goal of gaining mechanistic understanding of how this bacterium modulates colorectal carcinogenesis and metastasis (17). Given the similarities between enteric pathogen-mediated cytoskeletal reprogramming of the epithelium and the role of epithelial-mesenchymal plasticity in the etiopathogenesis of CRC, identifying virulence factors relevant to cellular cytoskeletal remodeling, as has been previously described with some foodborne human intestinal pathogens, is of particular interest for unraveling the mechanisms of CRC-associated bacteria (18, 19). Additionally, given that pathogens often produce many virulence factors, some of which work together in a coordinated fashion, it stands to reason that there may be co-occurrence relationships between well-studied virulence factors in fusobacteria and candidate virulence features. Such prior biological knowledge can inform analytical approaches for bacterial virulence factor discovery and prioritization of potential virulence features for subsequent study. Microbial genomic and pangenomic analysis are powerful tools that allow investigators to identify virulence gene signatures of specific bacterial strains in cancer (17, 20). Fic family proteins are encoded by a wide range of bacteria and secreted as toxins by specific bacterial pathogens (21). Herein, we analyze genomic variations in CRC isolates of *Fusobacterium* using a hypothesis-guided bioinformatic approach and delineate fusobacterial gene features relevant to CRC tumorigenesis and metastasis with a focus on Fic proteins.

## RESULTS

### Increased frequency of Fic domain-containing genes in Fap2^+^ fusobacterial genomes

Building on previous findings linking Fap2-mediated fusobacterial enrichment in tumors with CRC development and immune evasion (13, 22), we hypothesized that Fap2^+^ *Fusobacterium* colon-tumor isolates (CTIs) may harbor additional virulence features implicated in the molecular pathogenesis of CRC and tumorigenesis. To study the acquisition of potential virulence factors encoded by Fap2^+^ CTIs, we explored differences in the patterns of gene family presence or absence between Fap2^+^ CTI-1, 2, 6 and Fap2^–^ CTI-3, 5, 7 genomes (Fig. S1a). Using PPanGGOLiN’s expectation-maximization algorithm for partitioning bacterial gene families into optimal core and accessory subsets (see Materials and Methods), we found that Fap2^+^ CTIs encoded a relatively higher proportion of accessory genes compared to Fap2^–^ CTIs (Fig. S1b, left: Fisher’s exact test, Fap2^+ or –^ core vs Fap2^+ or –^ accessory genes, *P* = 7.50 × 10^−70^; Fig. S1b, right: Fap2^+^ accessory gene families, 53.71%), which was consistent with our overall observation that the genome sizes of Fap2^+^ CTIs (2.28 ~ 2.45 mbp) were relatively larger compared to Fap2^–^ CTIs (2.14 ~ 2.39 mbp).

**Fig 1:**
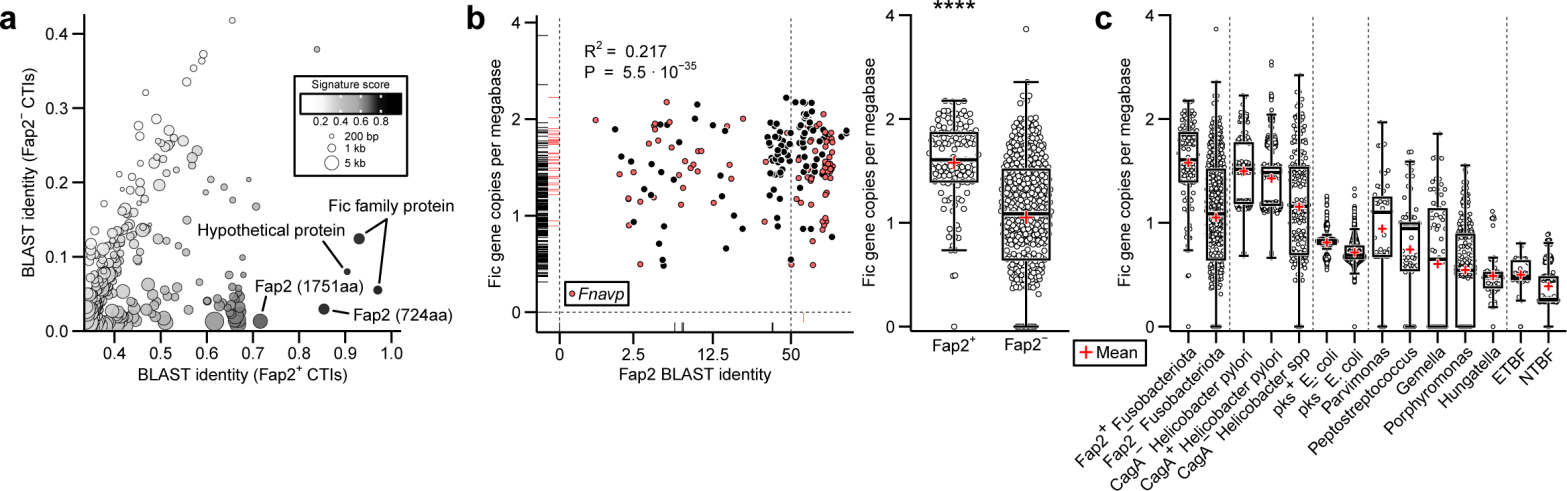
Quantitative pangenomic analysis identifies expansion of Fic gene families with Fap2^+^ fusobacterial strains. (**a**) K-mer-based subtractive genomic signature detection by Neptune’s algorithm using draft-level assemblies of *Fusobacterium* CTIs. Inclusion and exclusion genomes are Fap2^+^ *Fna* CTI-1, 2, 6 and Fap2^–^ CTI-3, 5, and 7, respectively. Neptune’s signature scores are a sum of BLAST identity-based sensitivity and specificity of a genomic signature matching inclusion genome regions. Each dot represents a bacterial genome fragment that contains open reading frame(s). (**b**) Analysis of genome length-adjusted Fic gene copy number from 622 publicly available fusobacterial genomes by Fap2 protein coverage (left, scatterplot; ~3 kbp Fap2 alignment length) or Fap2 genotype (right, boxplot). *Fnavp* (*F. nucleatum, animalis, vincentii, polymorphum*) in red and non-*Fnavp* species in black. (**c**) Abundance of Fic gene families in colorectal cancer-associated bacterial strains stratified by genus and species-level taxa. Enterotoxigenic *B. fragilis* (ETBF) strains are positive for fragilysin (*bft*) genes. Polyketide synthase-positive *Escherichia coli* (*pks*^+^ *E. coli*) strains are defined by the presence of one or more *clb* cluster genes (i.e., *clbA*, *clbB*, *clbS*, *clbQ*) in their genomes. Unless otherwise noted, minimum BLAST identity threshold for protein annotation in classifying bacterial genotype is 50%. NTBF, non-toxigenic *B. fragilis*. Plus symbols represent mean values.

To gain further insights into the genetic variations associated with Fap2^+^ CTIs, we performed a comparative k-mer search implemented in the Neptune subtractive sequence signature detection program (23). We identified genetic loci harboring protein open reading frames (ORFs) that contain conserved Fic motifs in Fap2^+^ CTIs (Fig. 1a), which have been reported to catalyze protein post-translational modifications via nucleotidyl-monophosphate transfer (NMPylation) reactions such as UMPylation, GMPylation, and AMPylation (24). We expanded our analysis of Fic domain containing (Fic family) genes to include 622 publicly available fusobacterial genomes. To perform gene co-occurrence analysis, we genotyped these strains using the blastp algorithm (*e*-value threshold of 10^−9^) for the presence of Fap2 homologs in combination with the Prokka pipeline’s whole-genome annotation and its hierarchical protein homolog search through a custom list of HMMER databases (25). For each fusobacterial genome, we then computed Fic family gene copy numbers normalized by genome sizes in Mbp as well as BLAST percent identities of Fap2 homologs. Using linear regression analysis, we found that normalized Fic gene copy numbers in fusobacterial genomes co-varied with Fap2 BLAST identities (Fig. 1b, scatterplot) as well as by Fap2 genotype (50% protein BLAST identity threshold; Fig. 1b, boxplot; Wilcoxon rank-sum test, *P* = 1.24 × 10^−20^).

Given that colonic intra-tumoral enrichment of Fusobacteriota in humans is well-established along with other gut-oral bacterial taxa (3, 4, 26), we asked if the genomic expansion of Fic family proteins is restricted to members of the Fusobacteriota and/or other gastrointestinal cancer-associated bacterial genera and species. We searched ORFs for Fic motifs in 6,685 bacterial strains belonging to the following taxa: *Helicobacter*, *Bacteroides fragilis*, *Escherichia coli*, *Peptostreptococcus*, *Parvimonas*, *Porphyromonas*, *Gemella*, and *Hungatella*. We genotyped strains of *Helicobacter* species, *B. fragilis*, and *E. coli* by the presence of well-known toxin-coding ORFs and ranked these taxa by the order of average normalized Fic gene copy number variations (Fig. 1c). We found that Fap2^+^ strains from Fusobacteriota have a propensity to harbor Fic family genes as compared to those from other cancer-associated taxa (Fig. 1c; Wilcoxon’s rank-sum test, Fap2^+^ Fusobacteriota vs all, *q* = 9.34 × 10^−56^ ~ 5.78 × 10^−3^). In addition, we annotated 50,553 draft and complete GenBank assemblies of core and pathogenic taxa from the human microbiome and confirmed that Fap2^+^ Fusobacteriota strains encoded one of the highest densities of Fic family proteins among human gut-associated bacterial strains (Fig. S2a).

### Metagenomic associations of fusobacterial Fic genes with locally advanced and metastatic Fap2^+^ colorectal tumors

**Fig 2:**
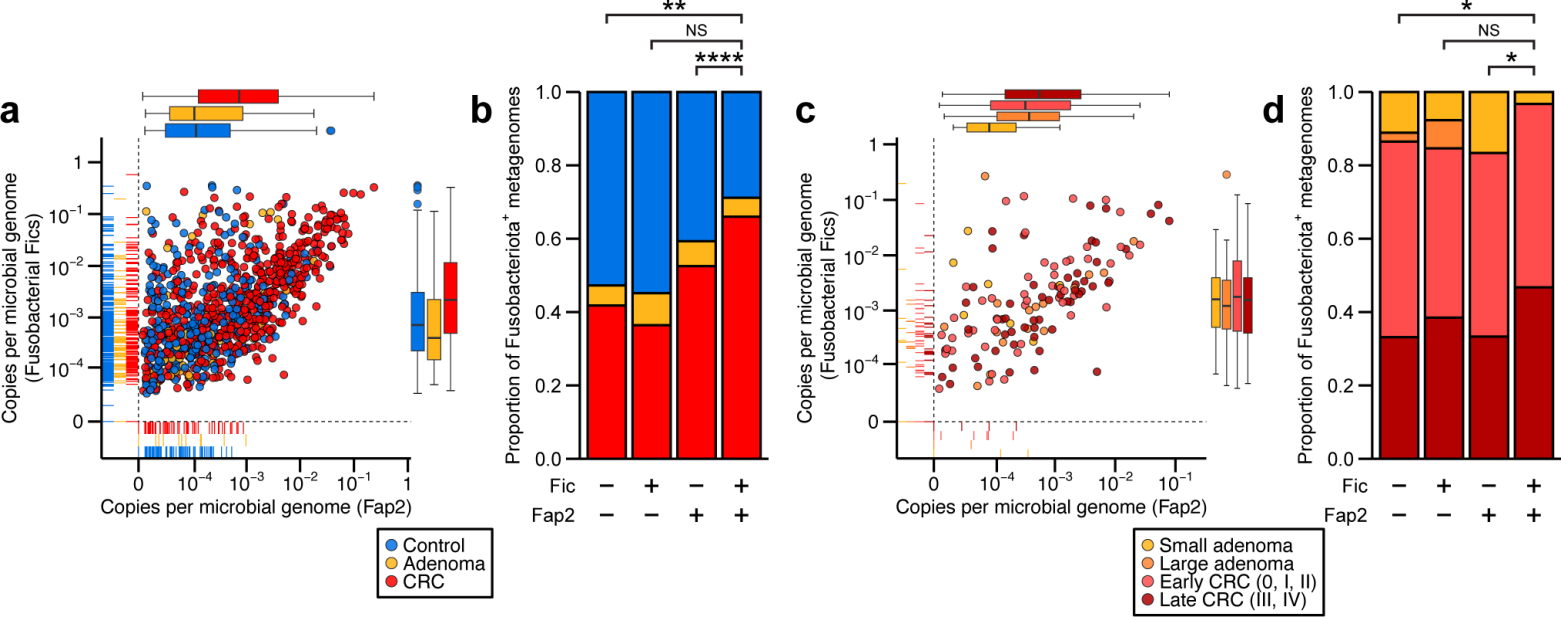
Metagenomic quantification of fusobacterial Fic gene families and Fap2 in patients with colorectal adenomas and adenocarcinomas. Linear regression analysis of Fusobacteriota taxon-specific abundance of Fic gene families and Fap2 in 2,088 fecal microbiomes by (**a**) case-control classes and (**c**) colorectal cancer tumor-node-metastasis (TNM) staging. Lines in left and bottom marginal plots represent metagenomes that are negative for either fusobacterial Fap2, Fic, or both. Metagenomic gene family abundance was normalized by sequencing depth and average microbial genome size. Analysis of fusobacterial Fic-Fap2 gene prevalence in clinical samples stratified by (**b**) diagnostic groups and (**d**) TNM stages. A positive sample is defined as having non-zero metagenomic abundance of a gene of interest. *P*-values from Fisher’s exact tests were adjusted by Benjamini-Hochberg (BH) step-up procedure; *q < 0.05; **q < 0.01; ***q < 0.001; ****q < 0.0001.

We next examined whether Fic gene family expansion is correlated with colorectal tumorigenesis in humans. We constructed a global cross-sectional shotgun metagenomic data set comprising non-redundant taxon-resolved fecal microbiome gene family profiles from patients diagnosed with colorectal adenomas and adenocarcinomas (*n*_control_ = 934; *n*_adenoma_ = 211; *n*_adenocarcinoma_ = 903) (3, 4, 26–35). Using this microbiome gene family abundance matrix, we tested if there were microbiome-wide differences in Fic family gene abundance but found no overall shift by case-control labels across studies (Fig. S3). Using a taxonomy-guided approach, we performed targeted abundance analysis of fusobacterial Fic gene families and Fap2 homologs, which revealed significant co-occurrence patterns in clinical samples (*R*^2,control^ = 0.144, *P* = 7.3 × 10^−35^; *R*^2,adenoma^ = 0.136, *P* = 2.1 × 10^−8^; *R*^2,CRC^ = 0.401, *P* = 2.3 × 10^−102^; Fig. 2a, rug scatterplot). In line with data from previous metagenomic marker gene surveys of CRC-associated gut microbiomes, our differential abundance analysis demonstrated a co-linear relationship of fusobacterial Fic and Fap2 genes in CRC consistent with our genome-based analysis (Fig. 2a, marginal boxplots). Focusing our analysis on Fusobacteriota mOTUs^+^ metagenomes (approximately 56.9%, 68.2%, and 28.3% of control-, adenoma-, and CRC-associated stool metagenomes, respectively, were negative for Fusobacteriota metagenomic Operational Taxonomic Units [mOTUs]), we determined that patients whose gut microbiomes are double-positive for fusobacterial Fic and Fap2 genes, possibly encoded by specific *Fusobacterium* species that frequently colonize CRC tumor tissues, are at an elevated risk of having CRC diagnoses not only at early but potentially also at late stages, relative to those that are negative for either one or both of these fusobacterial genes (Fig. 2b).

Probing the relationship between stool fusobacterial gene abundance and CRC tumor-lymph node-metastasis staging, we discovered that individuals diagnosed with late-stage cancers had a proportionally higher number of fusobacterial double-positive microbiomes than those at early stages (Fig. 2d; 46.8% and 79.0% of metagenomes associated with small and large adenomas, as well as 26.5% and 26.3% of metagenomes associated with early- and late-stage CRC, respectively, had no detectable abundance of Fusobacteriota mOTUs). Fecal abundance of fusobacterial Fap2 was relatively higher in patients diagnosed with premalignant or large adenomas as well as in patients with early- and late-stage adenocarcinomas compared to those with small adenomas (Fig. 2c, top marginal boxplot). Collectively, these observations may suggest a role for *Fusobacterium* in the neoplastic progression of malignant cancer cells distinct from mechanisms of fusobacterial Fap2-mediated binding to and signaling in tumor cells.

### Expansion of specific Fic gene families in Fap2^+^ *Fusobacterium animalis* (*Fa*) strains

**Fig 3:**
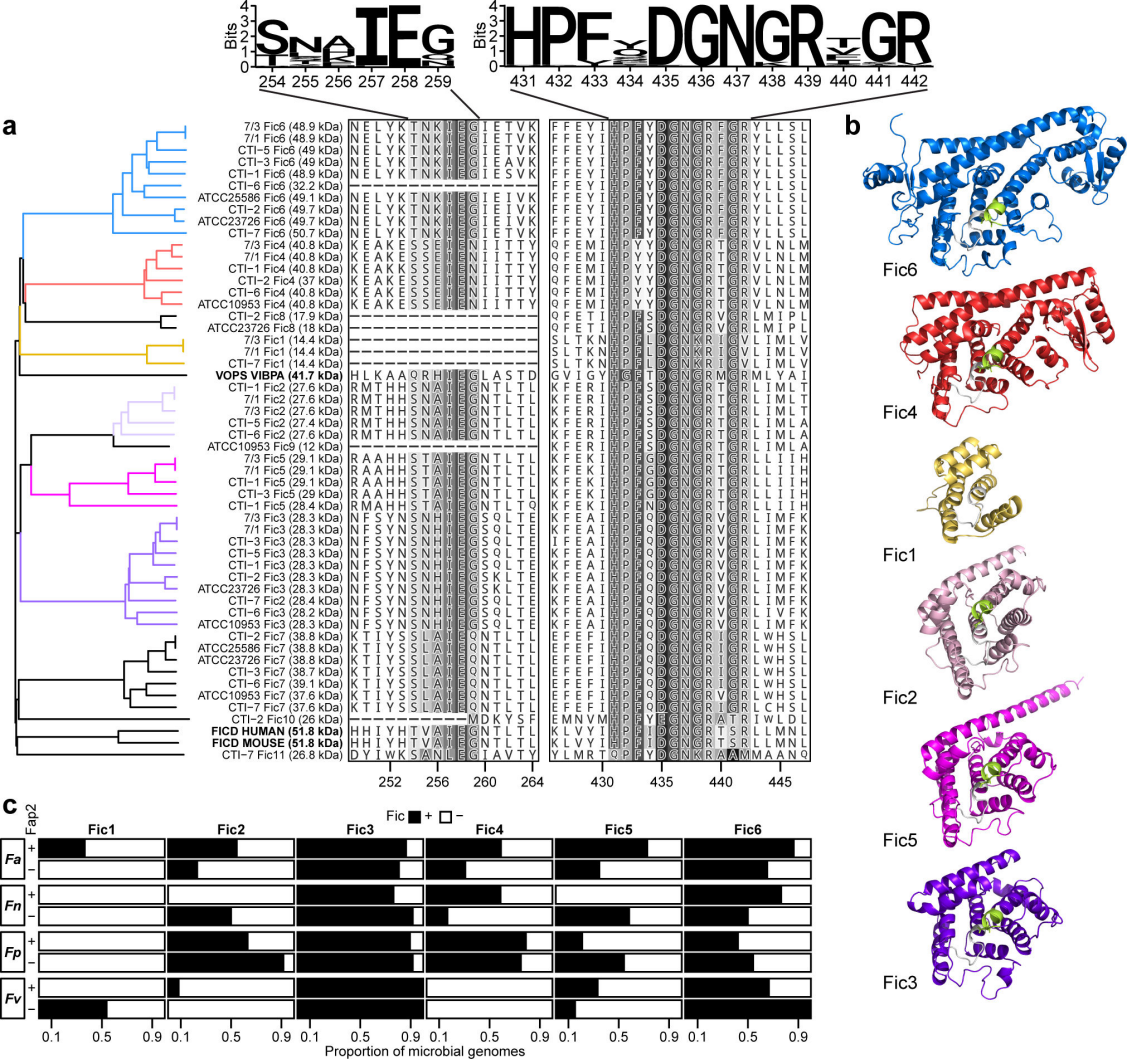
Characterization of Fic family proteins from *Fusobacterium* colon tumor isolates and type strains. (**a**) Clustal Omega alignment of Fic family proteins for identifications of conserved Fic motifs and their autoinhibitory domains. A phylogenetic tree was constructed using an identity matrix of aligned protein sequences and the *njs* function from the *ape* R package. VopS and FICD are protein adenylyltransferases from *V. parahaemolyticus* serotype O3:K6 and *Homo sapiens*/*Mus musculus*, respectively. *X*-axis denotes aligned amino acid coordinates. (**b**) AlphaFold2 structural predictions of six representative Fic enzymes encoded by *Fa*7/1 (39). Protein structures are assigned with corresponding clade colors as in (**a**). Green, autoinhibitory loop. Light-gray, Fic motif. (**c**) Prevalence of *Fa*7/1 Fic enzyme homologs having at least 50% BLAST identities in 146 publicly available *Fnavp* genomes stratified by Fap2 genotype. *Fa*, *F. animalis*; *Fn*, *Fusobacterium nucleatum*; *Fp*, *Fusobacterium polymorphum*; *Fv*, *Fusobacterium vincentii*.

To glean insights into Fic enzymes from *Fusobacterium* CTIs and select type strains, we characterized their functional domains by generating a multiple sequence alignment map and found several clades of fusobacterial Fic proteins with distinct motifs (Fig. 3a). Interestingly, none of these fusobacterial Fics were phylogenetic neighbors with the well-studied bacterial cytotoxic effector VopS from *Vibrio parahaemolyticus* nor the mammalian protein FIC domain protein adenylyltransferase (FICD) from humans and mice (18, 36, 37). The discovery of autoinhibitory sequence motifs, which have been shown to interact with Fic domains to block their catalytic activities, enabled classification of Fic enzymes into three distinct classes: class I with antitoxin, class II and III with autoinhibitory motifs (38). We observed that most *Fusobacterium* Fic enzymes belonged to class II or III with either N- or C-terminal α-helices (Fig. 3a) (38). Among those that lacked autoinhibitory motifs, e.g., Fic1, we located small hypothetical ORFs upstream of Fic gene ORFs (Fig. S4a) which may encode antitoxins with previously undescribed autoinhibitory residues that intermolecularly block catalytic activities of fusobacterial Fic domains. Consistent with the clade-level classification of Fic protein sequences, structural modeling with AlphaFold2 confirmed overall structural similarities between closely related Fic clade representatives (Fic proteins of *Fusobacterium animalis* 7/1, a strain with six representative Fic genes and previously described pro-tumorigenic properties, hereafter referred to as *Fa*7/1; Fig. 3b) (6, 39–42). Specifically, predicted protein structures showed the proper spatial positioning of active Fic ATP binding sites (light-gray, Fig. 3b) sterically hindered by autoinhibitory loops (yellow-green, Fig. 3b).

To study the population-level frequency of Fic gene representatives from Fap2^+^ *Fa*7/1 in *Fa*-related species*, nucleatum*, *vincentii*, *polymorphum* (*Fnavp*) (43), we used Prokka’s implementation of the BLAST algorithm to search for protein homologs at 50% BLAST identities in 146 draft and complete GenBank genomes of *Fnavp*. Among the *Fnavp* species analyzed, we found that, in general, the frequencies of Fic2, 4, and 5 in *Fa* were approximately twofold higher in Fap2^+^ than Fap2^–^ strains (Fig. 3c). The frequency of Fic4 nearly tripled in Fap2^+^ *Fn* compared to Fap2^–^ *Fn* strains. Differential prevalence of Fic5 was largely driven in part by Fap2 genotype across all four *Fnavp* species. Notably, Fic1 was frequently encoded by Fap2^+^ *Fa*. In contrast, Fic3 and 6 were present in at least 50% of all *Fnavp* species regardless of Fap2 positivity, suggesting that these two Fics, in particular, may have evolutionarily conserved functions in fusobacteria.

### Insertions and deletions of gene blocks encoding Fic family proteins are widespread among *Fusobacterium animalis* strains loci

**Fig 4:**
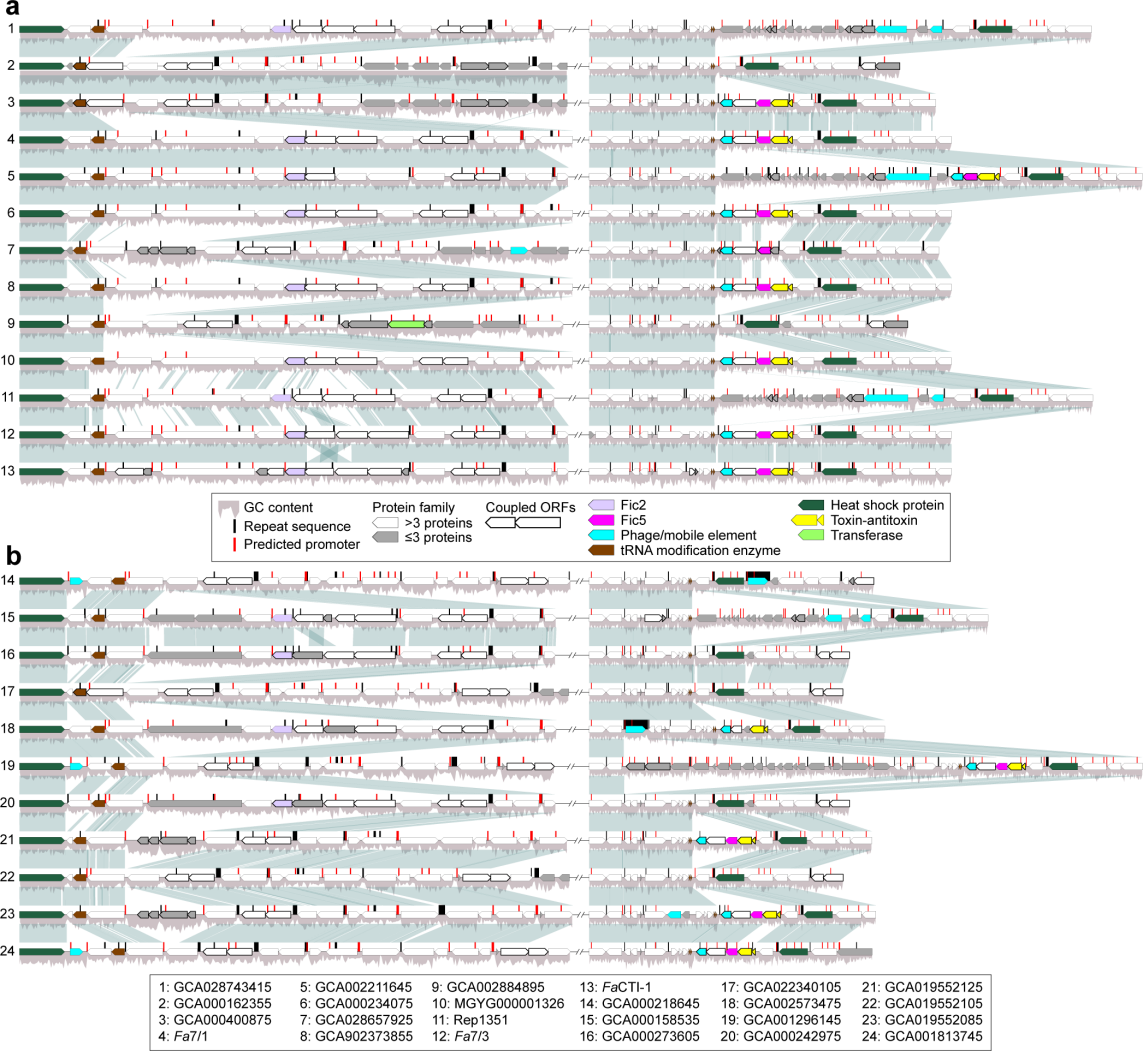
Genetic architecture and evolution of Fic gene loci in *Fusobacterium animalis* strains. Synteny analysis of gene blocks in Fic2 and Fic5 loci by minimap2 alignment of *Fa*7/1 Fic locus sequences against representative (**a**) Fap2^+^ and (**b**) Fap2^–^ *Fa* genomes. Locus sequences are genomic regions covering at least 10 kbp upstream and downstream of Fic genes. ORFs whose start and stop codons overlap or are within 5 bps apart are considered coupled ORFs. Repeat and promoter locus sequences were predicted by RepeatModeler2 and Promotech, respectively (44, 45). Locus-specific ORFs were clustered into protein families at 50% identity and coverage via MMseqs2 (46). Average percent GC was calculated over 50 bps sliding windows. Fic loci are segregated by line breaks per *Fa* strain.

Given the expansion we observed of specific Fic gene families in *Fa* genomes and previous data supporting *Fa* dominance in colorectal tumors (20), we next characterized the genetic architecture of Fic gene loci in *Fa* strains by mapping homologous regions of *Fa* genomes against *Fa*7/1 Fic gene loci. The presence or absence of Fic2 and 5 correlated with insertion or deletion of specific neighboring genes. Using the RepeatModeler2 pipeline (44), we observed overrepresentation of both intra- and inter-genic transposable element (TE) sequences in Fic1, 2, and 5 loci compared to Fic3, 4, and 6 (Fig. 4a; Fig. S4), and long tandem repeat sequences primarily in the inter-genic regions of most Fic loci (Fig. 4a; Fig. S4). With Promotech (45), we identified numerous intra- and inter-genic promoter sequences with high confidence, demonstrating that gene regulatory control in Fic loci regardless of Fap2 strain genotype may be as complex as any other genetic loci in *Fa*.

Furthermore, in Fic5 loci, phage/mobile elements and genomic islands of small ORFs were uniquely prevalent. Only Fic5 and 6 genes had adjacent pairs of toxin-antitoxin ORFs with heat shock proteins located either immediately upstream or downstream. tRNA modification enzymes were characteristic features of Fic2, 3, 4, and 6 loci. Fic1 loci were distinct in that the presence or absence of Fic1 genes was associated with rearrangement of large gene blocks upstream of Fic1 coordinates, which had potential bidirectional promoters (Fig. S4a and b). In Fic2 and 5 loci, there was an increased propensity toward Fic gene block rearrangement for regions that had either relatively high percent GC content or less well-conserved protein families. Overall, our synteny analysis uncovered elements of horizontal gene transfer and recombination events, which may drive the expansion and/or contraction of gene blocks associated with Fic family protein functions in Fap2^+^ *Fa*.

### Infection of murine colon adenocarcinoma tumorspheres by *Fusobacterium animalis* 7/1 upregulates fusobacterial Fic gene expression

**Fig 5:**
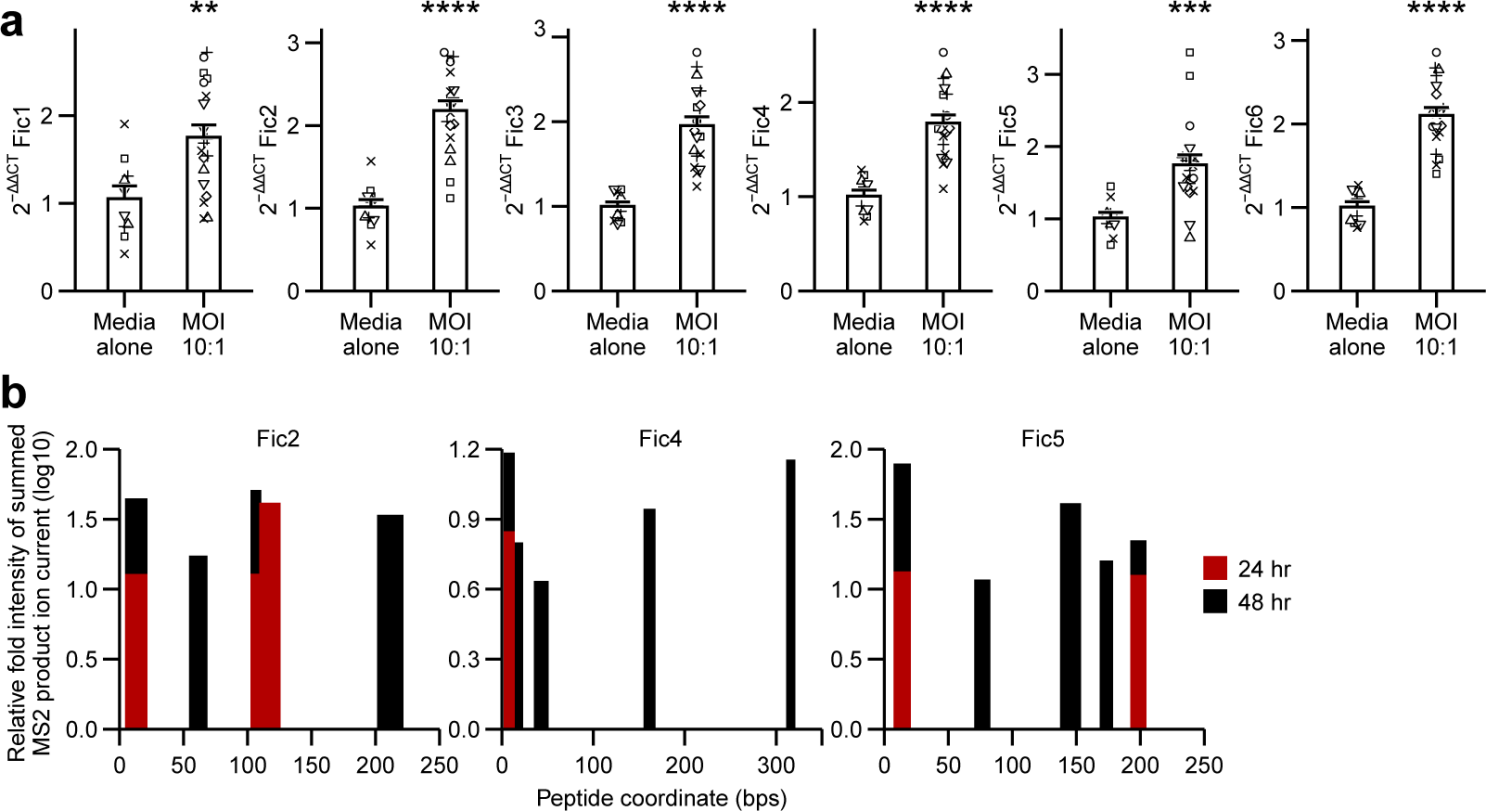
*Fa*7/1 Fic expression *in vitro.* (**a**) RT-qPCR analysis of *Fusobacterium* Fic gene expression at 6 h in *Fa*7/1 cocultures with Colon26 tumorspheres under anaerobic conditions. MOI, 10:1, *Fa*7/1 colony forming units (CFUs) to Colon26 cancer cells number. Relative gene expression values were normalized per experiment. Data represent seven independent experiments. Each symbol is one independent experiment. Error bars are SEM. *P*-values from Wilcoxon’s rank-sum tests were adjusted by Benjamini-Hochberg (BH) step-up procedure; *q < 0.05; **q < 0.01; ***q < 0.001; ****q < 0.0001. (**b**). Liquid chromatography tandem mass spectrometry-based proteomic detection of Fic family proteins in *Fa*7/1 monoculture supernatants from distinct growth phases. Ion peaks of peptides matching *Fa*7/1 Fic protein sequences for Fic2, 4, and 5.

To begin to determine environmental factors governing the gene regulation of *Fa*7/1 Fic loci in CRC, we analyzed gene expression of *Fa*7/1 exposed to tumorspheres under anaerobic conditions, which were generated from 96-h cultures of Colon26 cells, a murine colon adenocarcinoma cell line (47, 48). Although tumorspheres do not recapitulate the cellular heterogeneity and dynamic growth of *in vivo* tumors, studies have reported that they acquire metabolic phenotypes that align with those of primary and metastatic tumors (49, 50). After 6 h of *Fa*7/1 Colon26 anaerobic coculture (multiplicity of infection [MOI 10:1), we profiled the expression patterns of Fic1–6 by quantitative RT-PCR (RT-qPCR) relative to *Fa*7/1 overnight cultures re-grown in infection media and found significant differential gene upregulation by robust mean fold change analysis (RMFC, Fig. 5a); average RMFCs of Fic2, 3 and 6 were relatively higher (1.94–2.20, *q* = 4.49 × 10^−7^ ~ 4.27 × 10^−6^) than those of Fic1, 4, and 5 (1.70–1.78, *q* = 4.27 × 10^−6^ ~ 6.12 × 10^−3^).

Next, we sought to determine if Fic genes were expressed not only at the RNA level but also at the protein level. Given the complex mixture of proteins in bacteria-tumorsphere coculture supernatants, we transitioned to a simplified system for Fic protein detection using *Fa*7/1 supplemented tryptic soy broth monoculture supernatant. After analyzing mRNA expression of *Fa*7/1 Fic and virulence-associated genes across distinct growth phases (Fig. S5) (51), we selected 24- and 48-h timepoints for Fic peptide detection. Our proteomic analysis demonstrated the presence of fusobacterial peptides mapping to Fic2, 4, and 5 at 24- and 48-h culture timepoints (Fig. 5b) as well as fragments of FadA and Fap2 adhesins as has been previously described (Fig. S6a) (15). Our detection of Fic proteins produced by *Fa*7/1 is also supported by our reanalysis of publicly available proteomics data sets (Fig. S6b), wherein we found that fusobacterial Fic peptides were detectable in different fractions of *Fusobacterium* mono-, dual-, and multi-species cultures (Fig. S6c). In addition, changes in *Fa*7/1 gene expression in response to Colon26 tumorspheres were not exclusive to Fic family genes, as we observed alterations in several fusobacterial adhesins and virulence-associated genes (Fig. S7) (13, 17, 22, 43, 52). Overall, these data indicate that fusobacterial Fic genes are expressed at both the RNA and protein level, and their expression may be modulated by exposure to colon adenocarcinoma tumorspheres.

## DISCUSSION

In this work, we have integrated microbial genetic, genomic, metagenomic, and proteomic analyses of *Fusobacterium* species for virulence gene discovery relevant to colorectal carcinogenesis. Our analysis of *Fusobacterium* CTIs genomes reveals a potential role for fusobacterial Fic proteins in *Fusobacterium*-mediated colorectal tumorigenesis. Fic enzymes exhibit adenylyltransferase activities which transfer monophosphates, including but not limited to guanosine monophosphates, uridine monophosphates, and adenosine monophosphates (AMP; AMPylation), to specific protein residues (24). Bacterial pathogens, such as *V. parahaemolyticus, Legionella pneumophila,* and *Bartonella* spp., infect host eukaryotic cells by injecting Fic proteins via their types III, IV, and VI secretion systems (18, 53–55). AMPylation—addition of an AMP moiety from ATP onto the hydroxyl side chain of a target protein—for example, has been widely studied as a mechanism contributing to bacterial virulence and pathogenesis (38). These pathogens employ such cytosolic toxins or AMPylators (e.g., VopS) to modulate activities of host cytoskeleton-modifying enzymes, such as Rho GTPases (18, 56). However, the identification of, let alone production and export of, any such proteins by *Fusobacterium* species has been largely understudied, with a few exceptions (15, 57). Our study provides statistical evidence suggestive of virulence effectors associated with *Fusobacterium* Fap2-mediated enrichment and tumor potentiation in CRC. Mechanistically, the modulation of CRC tumorigenesis via post-translational modification of cancer cell proteome by toxins released from tumor-homing fusobacterial species may be biologically plausible and merits further investigation.

By targeted differential gene family abundance analysis using CRC-associated fecal microbiomes, we further confirmed co-enrichment of fusobacterial Fap2 and Fic gene families in clinical samples. Specifically, we discovered a trend toward increased prevalence of samples double-positive for fusobacterial Fap2 and Fic genes in late-stage CRC. Cancer cells that acquire a mesenchymal phenotype undergo numerous changes, including morphological ones, in a process known as epithelial-mesenchymal transition (EMT) (58). In CRC, EMT correlates with tumor invasiveness, metastatic potential, and resistance to treatment (59). The biochemical function of bacterial Fic enzymes has only been characterized in intestinal pathogens and opportunistic microbes, such as *Clostridioides difficile* and *Enterococcus faecalis* (60–62), and has yet to be studied in tumor resident bacteria. Fic enzymes encoded by invasive intracellular *Fusobacterium* species in cancer might have the potential to modulate dynamics of cytoskeletal remodeling and contribute to EMT. Epigenetic modifications also contribute to EMT (63, 64). *Coxiella burnetii* infection of stem cells and macrophages can result in reversible AMPylation of histone H3 (65). Such epigenetic modification is hypothesis generating for how bacterial Fic enzymes may contribute to cancer progression.

Our phylogenetic analysis indicated that fusobacterial Fic enzymes might have followed evolutionary pathways and trajectories distinct from those of known mammalian and bacterial pathogenic Fic enzymes. In *Fa* strains, known to be predominantly enriched in CRC tumor tissues (20), we identified expansions of specific Fic gene families located in genomic loci that had elements characteristic of horizontal gene transfer (HGT). Specifically, the presence or absence of Fic2 and 5 genes was associated with insertions and deletions of adjacent unidirectional overlapping genes, which may share a single promoter for coupled translation and expression (66). Although highly speculative, this could suggest that Fic2 and 5-containing gene blocks represent functionally coupled units of co-transcribing and/or co-adapting genes transferred through the evolution of *Fa* strains with host physiology, tissue pathology, and during tumorigenesis. The variable gene content observed in Fic5 loci harboring small ORF islands coupled with phage/mobile elements suggest prophage carriage in *Fa* (67, 68). The rearrangements of gene blocks associated with Fic5 genes may be linked to lifecycle regulation of lysogenic phages that contribute to strain competitiveness in response to host and tumor tissues-induced stress signaling pathways in polylysogenic *Fa* strains (67–71). Expansion of gene blocks containing Fic2 was associated with regional variations in GC content. Bacterial accessory genomes correlate with low GC content and high GC heterogeneity (72). Genomes of human gut-adapted anaerobes typically have heterogenous distributions of GC content relative to those of aerobes (73). *Fa*’s genomic evolution during oral-to-gut transmission in humans as it establishes a CRC niche is a process that varies considerably among individuals (74). Increased genetic alterations in Fic2 and 5 loci relative to other Fic gene loci in *Fa* strains may be reflective of *Fa*’s HGT-driven adaptation along the human oral-gastrointestinal tract.

Using *in vitro* bacteria-tumorsphere cocultures, we began to explore what factors may regulate Fic gene expression in a clinical *Fa* isolate that adheres to and invades cancer cells and promote colonic tumorigenesis *in vivo* (6, 75). Our RT-qPCR analysis of bacterial transcripts in these cocultures indicates exposure of fusobacteria to cancer cells correlates with increased fusobacterial Fic gene expression, providing a glimpse into the host-fusobacteria cross-talk in carcinogenesis. Using proteome profiling, we found a detectable and non-negligible amount of Fic peptides in *Fa* monoculture supernatant potentially suggestive of Fic protein secretion. These data supporting *in vitro* Fic expression by *Fa* in response to cancer cells, as well as during monoculture growth, point to potential mechanisms of interactions between fusobacterial Fic proteins and colon adenocarcinoma cells. Overall, our bioinformatic analyses together with our *in vitro* findings shine a light on Fic proteins, which warrant investigation in fusobacterial CRC etiopathogenesis.

Fusobacterial species are among a growing number of bacteria that are enriched in adenomas, colorectal cancers, and their metastases (76–78). Unlike colibactin-producing bacteria such as some strains of *E. coli,* fusobacteria do not harbor DNA damaging genotoxins, nor do fusobacteria harbor a metalloprotease like *Bacteroides fragilis* toxin that can increase colonic epithelial cell (CEC) proliferation, suppress CEC apoptosis, and induce CEC epigenetic alterations, which can lead to DNA damage (79). While the fusobacterial adhesins, Fap2, FadA, and RadD, all seem to enhance tumorigenesis in preclinical tumor models via a multiplicity of signaling pathways that contribute to tumorigenesis (78–80), questions still remain whether fusobacteria are truly oncogenic. A highly regarded and current conceptualization of carcinogenesis by Hanahan includes eight hallmarks of cancer: “acquired capabilities for sustaining proliferative signaling, evading growth suppressors, resisting cell death, enabling replicative immortality, inducing/accessing vasculature, activating invasion and metastasis, reprogramming cellular metabolism, and avoiding immune destruction” (81). Many microbes possess these characteristics themselves and some bacteria, such as certain fusobacterial species, can confer these features on an evolving colon tumor in preclinical models. Limitations in our knowledge of fusobacterial virulence factors and how fusobacteria may contribute to oncogenesis motivated this investigation, and further studies are needed to unravel how fusobacteria and other tumor-associated bacteria may contribute to the hallmarks of cancer.

## MATERIALS AND METHODS

### Microbial pangenomics and metagenomics database construction

GenBank assembly reports and taxonomy databases for bacterial genomes of interest were retrieved using the taxizedb package in R (last accessed on 24 March 2024; see Table S1 for fusobacterial genomes). We included non-redundant high-quality metagenome-assembled (MAGs) and isolate genomes from several human gut culturomics and reference microbiome genome catalog studies (RMGC; PRJNA482748, PRJNA903559, PRJDB9057, PRJNA544527, and other download links available through study-specific repositories) (82–88). Microbial genomes were annotated using the Prokka pipeline with additional hierarchical search databases (*e*-value = 10^−9^; protein coverage = 0.8) comprising BLASTp reference sequences (first pass) of Fap2, bft (89), and clb cluster genes (90–92), and hidden Markov model (HMM) profiles of built-in HAMAP (release 2022_03; second pass) and the protein family database Pfam (v.35; third pass) (25, 93, 94). Bacterial Fic genes were identified by matching accession numbers from Pfam (PF02661) and Clusters of Orthologous Genes (COG) (COG3177, COG2184) and UniProt (Q9K0V1). The GTDB-Tk toolkit (v.2.1.0) was used to classify microbial genomes based on the Genome Taxonomy Database tree (release 207_v2) (95, 96). mOTUs database was built with 18,018 isolate genomes and MAGs from the RMGC database using the mOTUs extender (97), resulting in 442,317 single-copy marker genes and 6,343 mOTUs species.

### Quantification of microbiome gene family abundance

Metagenomic reads from human fecal microbiomes, drawn from the following CRC microbiome studies (3, 4, 26–35), were quality-trimmed using the Trimmomatic program (v.0.39; options, illumina_adapters:2:36:7:1:TRUE leading:3 trailing:3 slidingwindow:4:15 minlen:36 tophred33) (98). Reads mapping to human chromosomes (CHM13v2) and sequencing vector library (UniVec and EmVec) were removed using BWA-MEM (v.0.7.17-r1188), Samtools (v.1.17), and Picard (v.2.27.5) (99–102). Quality-controlled reads were aligned against a non-redundant microbial gene family catalog, which was created by MMseqs2 clustering (October 2023 release; options: --min-seq-id 1, -c 1, --cov-mode 0) of CDS nucleotide sequences from the RMGC genome collection and CTIs Fap2 homologs from 622 GenBank fusobacterial genomes (available as of 24 March 2024) annotated by the Prokka pipeline (46). Gene family abundance was quantified using msamtools subcommands (filter options: -p 95 -z 50 --besthit, profile options: --multi=proportional—unit --unit=ab) and normalized to obtain copies per microbial genome by incorporating average genome size estimates from the MicrobeCensus (v.1.1.0), as previously described (103–105). We calculated genome length-adjusted units to describe Fic gene copy number variations instead of absolute metrics due to gene counts biases toward larger bacterial genomes (Fig. S2b).

### Comparative genomics and genetics

We used Prokka’s genome annotation data for *Fusobacterium* colon tumor isolates (CTIs-1, 2, 3, 5, 6, 7 with previously defined Fap2 phenotype [13]; see Table S1 for GenBank fusobacterial genome accession numbers) to classify core and accessory genes via the PPanGGOLiN approach (cluster options: --identity 0.9 --coverage 0.9 --mode 1) (106). PPanGGOLiN’s gene presence-absence matrix and the pangenome graphs were visualized using ape (njs function), ggtree packages in R (107, 108), and the Gephi platform (ForceAtlas2 layout, scaling: 5,000–20,000, stronger gravity: yes, gravity: 2–5, edge weight influence: 3–5) (109), respectively. Genomic signature sequences specific to a group of Fap2^+^ CTIs were detected using the Neptune’s subtractive k-mer matching and aggregate BLAST scoring algorithms (options: --filter-length 0.5 --filter-percent 0.5) (23). Fifty percent BLAST identity was used to determine the presence of Fap2 homologs in fusobacterial genomes, as it sufficiently covers autotransporter domains of Fap2 (~1.5–1.6 kbp of ~3 kbp Fap2 protein sequence) and has been previously shown to be a threshold below which functional divergence in proteins rapidly increases (110, 111). Sequence motif analysis was performed via multiple sequence alignment of Fic family proteins from CTIs and *Fusobacterium* type strains using the ClustalOmega algorithm implemented in the msa R/Bioconductor package (112). Sequence heatmap and logos were generated using the ggmsa and ggseqlogo packages in R (113, 114). Fic proteins from *Fnavp* genomes were filtered by BLAST identity scores at 50% threshold against *Fa*7/1 Fic1–6 for differential prevalence analysis. Protein structures of Fic enzymes were predicted using the ColabFold software, a graphics processing unit (GPU)-accelerated AlphaFold2 combined with MMseqs2 homology search, and visualized in open-source PyMOL (v.3.0.0) (39, 40).

### Genomic locus characterization and synteny

Locus sequences of *Fa*7/1 covering at least 10 kbp upstream and downstream of Fic genes families were extracted using BEDTools and mapped against homologous regions of *Fnavp* genomes using the Minimap2 aligner (v.2.21-r1071, options: -c -P -z5).^115^, ^116^ Pairwise mapping format (PAF) data from Minimap2 aligner were imported with the parser function from pafr package and visualized using gggenomes package in R. Average GC content was computed across 50 bps non-overlapping sliding windows using the Biostrings R/Bioconductor package. ORFs were considered to be coupled if the start and stop codons overlap or are within 5 bps apart. MMseqs2 (options: --min-seq-id 0.5, -c 0.5, --cov-mode 0) clustering of locus-specific ORFs was performed to annotate less well-conserved protein families. SeqKit (v.2.6.0; sliding options -s 1 -W 40) was used to generate overlapping k-mer sequences (k = 40; k – 1) (117), which served as input to Promotech (v.1.0; options: -s -m RF-HOT) for the prediction of locus-specific promoters (high-confidence probability cutoff = 0.8) (45). Low-complexity, tandem, or TE repeat sequences were annotated using RepeatModeler2 with default options (44).

### Quantitative RT-PCR gene expression analysis of bacteria-tumorsphere cocultures

Murine colon adenocarcinoma cells (Colon26) were seeded in v-bottom 96-well plates (50,000 cells per well; Cepham Life Sciences, Fulton, MD) precoated with anti-adherent solution (STEMCELL Technologies, Cambridge, MA) and grown for 4 days in RPMI 1640 media supplemented with GlutaMAX (Thermo Fisher Scientific, Waltham, MA) and 10% standard-filtered fetal bovine serum (FBS) (Sigma-Aldrich, St. Louis, MO) without antibiotics. *Fa*7/1 was grown in filter-sterilized tryptic soy broth with hemin (5 µg/mL) and menadione (1 µg/mL) at 37°C under anaerobic conditions in a vinyl chamber (Coy Lab Products, Grass Lake, MI) (41). Tumorspheres (approximately 2.5–3.0 × 10^6^ viable cells) were rinsed three times with phenol red-free RPMI 1640 and infected with *Fa*7/1 at multiplicity of infection of 10:1 (colony forming units or CFUs to cancer cells number) in pre-reduced phenol red-free RPMI 1640 supplemented with L-glutamine in 15 mL Falcon tubes for 6 h at 37°C under anaerobic conditions.

To enrich microbial RNA, tumorspheres were pre-treated with 0.0125% saponin in Tris-buffered saline as previously described (118), and centrifuged at 5,000 *g* for 15 min to deplete cell-free RNA. Total RNA was then isolated using QIAzol Lysis Reagent in combination with Max Bacterial Enhancement Reagent (Thermo Fisher Scientific, Waltham, MA) and purified using the Direct-zol RNA Miniprep Kit with on-column DNA digestion (Zymo Research, Irvine, CA) followed by double DNAse treatment with TURBO DNA-free Kit (Thermo Fisher Scientific, Waltham, MA). Complementary DNA (cDNA) was synthesized from 5 µg RNA using Maxima H Minus cDNA Synthesis Master Mix (Thermo Fisher Scientific, Waltham, MA) and subjected to RT-qPCR analysis (40 ng per technical duplicate) using the KAPA SYBR FAST Universal Kit (Roche) on an Agilent Mx3005P cycler. Pan-*Fusobacterium* species-specific primers were designed using the PrimerQuest Tool to target conserved regions of gene sequence templates that have high coverage across *Fnavp* genomes as assessed by the Prider package in R (119). Specificities of primer sequences were checked against the nt database using the Primer-BLAST algorithm (120), and were further validated using *Fusobacterium* spike-in control DNA samples. Ct values were normalized per experiment using the geometric mean of eubacterial *16S*, fusobacterial *rpoB* and *recA* internal control genes relative to *Fa*7/1 overnight culture inoculum (16 ~ 24 h; ~10^8^ CFUs) in infection media (121). Robust mean fold change was calculated by determining the average of all combinatorial pairs of fold change values that fell between 20th and 80th percentile range (122). Primers used in this study are listed in Table S2.

### Proteomic analysis of bacterial monoculture secretome

Supernatants of *Fa*7/1 monocultures from 24- and 48-h growth timepoints were filter-sterilized (100 mL; pore size of 0.22 µm), concentrated with Amicon Ultra Centrifugal 10 kDA Filter (Sigma-Aldrich, St. Louis, MO), and precipitated with methanol/chloroform (4:1) followed by solubilization in 8M urea. Proteins were analyzed on an SDS-PAGE gel visualized with QC Colloidal Coomassie Stain (Bio-Rad, Waltham, MA), and bands corresponding to predicted protein sizes of *Fa*7/1 Fic1-6 were excised and submitted to the Harvard Center for Mass Spectrometry for peptide detection on a Q Exactive HF-X Hybrid Quadrupole-Orbitrap mass spectrometry (MS) system (Thermo Fisher Scientific, Waltham, MA).

Raw MS data from the Thermo Orbitrap instrument were converted to mzML files using the ThermoRawFileParser (v.1.4.3; options: -f = 1 -m = 1) (123). Non-redundant fusobacterial pan-proteomics database was created via MMseqs2 clustering of protein ORFs from 622 fusobacterial genomes (options: --min-seq-id 1, -c 1, --cov-mode 0). Peptide identification from mzML data were performed using the MS-GF + MS/MS proteomics database search tool (Release 20230112; options: -e 1 -inst 3 -m 3 -tda 1) with specification for static modification (carbamidomethyl C) and dynamic modifications (oxidation M, variable carbamidomethyl N-term, and acetylation protein N-term) (124). MS/MS identification data (mzid) were quality-filtered by estimating false discovery rates (FDR) of peptide identifications using the MSnID R/Bioconductor package. Ion peaks data from peptide-spectrum matches (FDR < 0.05) were normalized relative to minimum peptide ion current intensities per proteome and visualized using the ggprotein function in ggcoverage package in R (125). Publicly available *Fusobacterium* proteomes (PXD004888, PXD008288, PXD008444, PXD037520) from the ProteomeXchange database were reanalyzed accordingly (126–130).

## SUPPLEMENTAL MATERIAL

Fig. S1 mbio.03732-24-s0001.tif PPanGGOLiN analysis of *Fusobacterium* CTIs.

Fig. S2 mbio.03732-24-s0002.tif Taxon-specific variations in Fic gene copy number in 50,553 microbial genomes.

Fig. S3 mbio.03732-24-s0003.tif Taxon-agnostic abundance of Fic genes in the global CRC gut microbiome cohort.

Fig. S4 mbio.03732-24-s0004.tif Genetic architectures of conserved Fic gene loci in *Fusobacterium animalis* strains.

Fig. S5 mbio.03732-24-s0005.tif Fa7/1 gene expression from distinct monoculture growth phases.

Fig. S6 mbio.03732-24-s0006.tif Proteomic analysis of fusobacterial culture samples.

Fig. S7 mbio.03732-24-s0007.tif Expression of genes associated with fusobacterial Fic loci and virulence in Fa7/1 Colon26 cocultures at 6 h under anaerobic conditions.

Table S1 mbio.03732-24-s0008.xlsx Fusobacterial genomes used in this study.

Table S2 mbio.03732-24-s0009.xlsx Primers used in this study.

ASM does not own the copyrights to Supplemental Material that may be linked to, or accessed through, an article. The authors have granted ASM a non-exclusive, world-wide license to publish the Supplemental Material files. Please contact the corresponding author directly for reuse.

## References

[1] Castellarin M, Warren RL, Freeman JD, Dreolini L, Krzywinski M, Strauss J, Barnes R, Watson P, Allen-Vercoe E, Moore RA, Holt RA. 2012. Fusobacterium nucleatum infection is prevalent in human colorectal carcinoma. Genome Res 22:299–306. doi:10.1101/gr.126516.111 22009989 PMC3266037

[2] Kostic AD, Gevers D, Pedamallu CS, Michaud M, Duke F, Earl AM, Ojesina AI, Jung J, Bass AJ, Tabernero J, Baselga J, Liu C, Shivdasani RA, Ogino S, Birren BW, Huttenhower C, Garrett WS, Meyerson M. 2012. Genomic analysis identifies association of Fusobacterium with colorectal carcinoma. Genome Res 22:292–298. doi:10.1101/gr.126573.111 22009990 PMC3266036

[3] Wirbel J, Pyl PT, Kartal E, Zych K, Kashani A, Milanese A, Fleck JS, Voigt AY, Palleja A, Ponnudurai R, et al.. 2019. Meta-analysis of fecal metagenomes reveals global microbial signatures that are specific for colorectal cancer. Nat Med 25:679–689. doi:10.1038/s41591-019-0406-6 30936547 PMC7984229

[4] Thomas AM, Manghi P, Asnicar F, Pasolli E, Armanini F, Zolfo M, Beghini F, Manara S, Karcher N, Pozzi C, et al.. 2019. Metagenomic analysis of colorectal cancer datasets identifies cross-cohort microbial diagnostic signatures and a link with choline degradation. Nat Med 25:667–678. doi:10.1038/s41591-019-0405-7 30936548 PMC9533319

[5] GBD 2019 Colorectal Cancer Collaborators. 2022. Global, regional, and national burden of colorectal cancer and its risk factors, 1990-2019: a systematic analysis for the Global Burden of Disease Study 2019. Lancet Gastroenterol Hepatol 7:627–647. doi:10.1016/S2468-1253(22)00044-9 35397795 PMC9192760

[6] Kostic AD, Chun E, Robertson L, Glickman JN, Gallini CA, Michaud M, Clancy TE, Chung DC, Lochhead P, Hold GL, El-Omar EM, Brenner D, Fuchs CS, Meyerson M, Garrett WS. 2013. Fusobacterium nucleatum potentiates intestinal tumorigenesis and modulates the tumor-immune microenvironment. Cell Host Microbe 14:207–215. doi:10.1016/j.chom.2013.07.007 23954159 PMC3772512

[7] Bullman S, Pedamallu CS, Sicinska E, Clancy TE, Zhang X, Cai D, Neuberg D, Huang K, Guevara F, Nelson T, et al.. 2017. Analysis of Fusobacterium persistence and antibiotic response in colorectal cancer. Science 358:1443–1448. doi:10.1126/science.aal5240 29170280 PMC5823247

[8] Holt RA. 2023. Oncomicrobial vaccines: the potential for a Fusobacterium nucleatum vaccine to improve colorectal cancer outcomes. Cell Host Microbe 31:141–145. doi:10.1016/j.chom.2022.11.014 36634619

[9] Mima K, Sukawa Y, Nishihara R, Qian ZR, Yamauchi M, Inamura K, Kim SA, Masuda A, Nowak JA, Nosho K, Kostic AD, Giannakis M, Watanabe H, Bullman S, Milner DA, Harris CC, Giovannucci E, Garraway LA, Freeman GJ, Dranoff G, Chan AT, Garrett WS, Huttenhower C, Fuchs CS, Ogino S. 2015. Fusobacterium nucleatum and T cells in colorectal carcinoma. JAMA Oncol 1:653–661. doi:10.1001/jamaoncol.2015.1377 26181352 PMC4537376

[10] Zheng D-W, Dong X, Pan P, Chen K-W, Fan J-X, Cheng S-X, Zhang X-Z. 2019. Phage-guided modulation of the gut microbiota of mouse models of colorectal cancer augments their responses to chemotherapy. Nat Biomed Eng 3:717–728. doi:10.1038/s41551-019-0423-2 31332342

[11] Parhi L, Alon-Maimon T, Sol A, Nejman D, Shhadeh A, Fainsod-Levi T, Yajuk O, Isaacson B, Abed J, Maalouf N, Nissan A, Sandbank J, Yehuda-Shnaidman E, Ponath F, Vogel J, Mandelboim O, Granot Z, Straussman R, Bachrach B. 2020. Breast cancer colonization by Fusobacterium nucleatum accelerates tumor growth and metastatic progression. Nat Commun 11:3259. doi:10.1038/s41467-020-16967-2 32591509 PMC7320135

[12] Xu M, Yamada M, Li M, Liu H, Chen SG, Han YW. 2007. FadA from Fusobacterium nucleatum utilizes both secreted and nonsecreted forms for functional oligomerization for attachment and invasion of host cells. J Biol Chem 282:25000–25009. doi:10.1074/jbc.M611567200 17588948

[13] Abed J, Emgård JEM, Zamir G, Faroja M, Almogy G, Grenov A, Sol A, Naor R, Pikarsky E, Atlan KA, Mellul A, Chaushu S, Manson AL, Earl AM, Ou N, Brennan CA, Garrett WS, Bachrach G. 2016. Fap2 Mediates Fusobacterium nucleatum colorectal adenocarcinoma enrichment by binding to tumor-expressed Gal-GalNAc. Cell Host Microbe 20:215–225. doi:10.1016/j.chom.2016.07.006 27512904 PMC5465824

[14] Rubinstein MR, Wang X, Liu W, Hao Y, Cai G, Han YW. 2013. Fusobacterium nucleatum promotes colorectal carcinogenesis by modulating E-cadherin/β-catenin signaling via its FadA adhesin. Cell Host Microbe 14:195–206. doi:10.1016/j.chom.2013.07.012 23954158 PMC3770529

[15] Meng Q, Gao Q, Mehrazarin S, Tangwanichgapong K, Wang Y, Huang Y, Pan Y, Robinson S, Liu Z, Zangiabadi A, Lux R, Papapanou PN, Guo XE, Wang H, Berchowitz LE, Han YW. 2021. Fusobacterium nucleatum secretes amyloid-like FadA to enhance pathogenicity. EMBO Rep 22:e52891. doi:10.15252/embr.202152891 34184813 PMC8406402

[16] Parhi L, Abed J, Shhadeh A, Alon-Maimon T, Udi S, Ben-Arye SL, Tam J, Parnas O, Padler-Karavani V, Goldman-Wohl D, Yagel S, Mandelboim O, Bachrach G. 2022. Placental colonization by Fusobacterium nucleatum is mediated by binding of the Fap2 lectin to placentally displayed Gal-GalNAc. Cell Rep 38:110537. doi:10.1016/j.celrep.2022.110537 35320712

[17] Ma X, Sun T, Zhou J, Zhi M, Shen S, Wang Y, Gu X, Li Z, Gao H, Wang P, Feng Q. 2023. Pangenomic study of Fusobacterium nucleatum reveals the distribution of pathogenic genes and functional clusters at the subspecies and strain levels. Microbiol Spectr 11:e05184-22. doi:10.1128/spectrum.05184-22 37042769 PMC10269558

[18] Yarbrough ML, Li Y, Kinch LN, Grishin NV, Ball HL, Orth K. 2009. AMPylation of Rho GTPases by Vibrio VopS disrupts effector binding and downstream signaling. Science 323:269–272. doi:10.1126/science.1166382 19039103

[19] Lu J, Kornmann M, Traub B. 2023. Role of epithelial to mesenchymal transition in colorectal cancer. Int J Mol Sci 24:14815. doi:10.3390/ijms241914815 37834263 PMC10573312

[20] Zepeda-Rivera M, Minot SS, Bouzek H, Wu H, Blanco-Míguez A, Manghi P, Jones DS, LaCourse KD, Wu Y, McMahon EF, Park S-N, Lim YK, Kempchinsky AG, Willis AD, Cotton SL, Yost SC, Sicinska E, Kook J-K, Dewhirst FE, Segata N, Bullman S, Johnston CD. 2024. A distinct Fusobacterium nucleatum clade dominates the colorectal cancer niche. Nature New Biol 628:424–432. doi:10.1038/s41586-024-07182-w PMC11006615 38509359

[21] Veyron S, Peyroche G, Cherfils J. 2018. FIC proteins: from bacteria to humans and back again. Pathog Dis 76. doi:10.1093/femspd/fty012 29617857

[22] Gur C, Ibrahim Y, Isaacson B, Yamin R, Abed J, Gamliel M, Enk J, Bar-On Y, Stanietsky-Kaynan N, Coppenhagen-Glazer S, Shussman N, Almogy G, Cuapio A, Hofer E, Mevorach D, Tabib A, Ortenberg R, Markel G, Miklić K, Jonjic S, Brennan CA, Garrett WS, Bachrach G, Mandelboim O. 2015. Binding of the Fap2 protein of Fusobacterium nucleatum to human inhibitory receptor TIGIT protects tumors from immune cell attack. Immunity 42:344–355. doi:10.1016/j.immuni.2015.01.010 25680274 PMC4361732

[23] Marinier E, Zaheer R, Berry C, Weedmark KA, Domaratzki M, Mabon P, Knox NC, Reimer AR, Graham MR, Chui L, Patterson-Fortin L, Zhang J, Pagotto F, Farber J, Mahony J, Seyer K, Bekal S, Tremblay C, Isaac-Renton J, Prystajecky N, Chen J, Slade P, Van Domselaar G. 2017. Neptune: a bioinformatics tool for rapid discovery of genomic variation in bacterial populations. Nucleic Acids Res 45:e159–e159. doi:10.1093/nar/gkx702 29048594 PMC5737611

[24] Garcia-Pino A, Zenkin N, Loris R. 2014. The many faces of Fic: structural and functional aspects of Fic enzymes. Trends Biochem Sci 39:121–129. doi:10.1016/j.tibs.2014.01.001 24507752

[25] Seemann T. 2014. Prokka: rapid prokaryotic genome annotation. Bioinformatics 30:2068–2069. doi:10.1093/bioinformatics/btu153 24642063

[26] Yachida S, Mizutani S, Shiroma H, Shiba S, Nakajima T, Sakamoto T, Watanabe H, Masuda K, Nishimoto Y, Kubo M, et al.. 2019. Metagenomic and metabolomic analyses reveal distinct stage-specific phenotypes of the gut microbiota in colorectal cancer. Nat Med 25:968–976. doi:10.1038/s41591-019-0458-7 31171880

[27] Gupta A, Dhakan DB, Maji A, Saxena R, P.k. VP, Mahajan S, Pulikkan J, Kurian J, Gomez AM, Scaria J, Amato KR, Sharma AK, Sharma VK. 2019. Association of flavonifractor plautii, a flavonoid-degrading bacterium, with the gut microbiome of colorectal cancer patients in India. mSystems 4. doi:10.1128/msystems.00438-19 PMC7407896 31719139

[28] Vogtmann E, Hua X, Zeller G, Sunagawa S, Voigt AY, Hercog R, Goedert JJ, Shi J, Bork P, Sinha R. 2016. Colorectal cancer and the human gut microbiome: reproducibility with whole-genome shotgun sequencing. PLoS One 11:e0155362. doi:10.1371/journal.pone.0155362 27171425 PMC4865240

[29] Yang Y, Du L, Shi D, Kong C, Liu J, Liu G, Li X, Ma Y. 2021. Dysbiosis of human gut microbiome in young-onset colorectal cancer. Nat Commun 12:6757. doi:10.1038/s41467-021-27112-y 34799562 PMC8604900

[30] Yang J, Li D, Yang Z, Dai W, Feng X, Liu Y, Jiang Y, Li P, Li Y, Tang B, Zhou Q, Qiu C, Zhang C, Xu X, Feng S, Wang D, Wang H, Wang W, Zheng Y, Zhang L, Wang W, Zhou K, Li S, Yu P. 2020. Establishing high-accuracy biomarkers for colorectal cancer by comparing fecal microbiomes in patients with healthy families. Gut Microbes 11:918–929. doi:10.1080/19490976.2020.1712986 31971861 PMC7524397

[31] Feng Q, Liang S, Jia H, Stadlmayr A, Tang L, Lan Z, Zhang D, Xia H, Xu X, Jie Z, et al.. 2015. Gut microbiome development along the colorectal adenoma-carcinoma sequence. Nat Commun 6:6528. doi:10.1038/ncomms7528 25758642

[32] Zeller G, Tap J, Voigt AY, Sunagawa S, Kultima JR, Costea PI, Amiot A, Böhm J, Brunetti F, Habermann N, et al.. 2014. Potential of fecal microbiota for early-stage detection of colorectal cancer. Mol Syst Biol 10:766. doi:10.15252/msb.20145645 25432777 PMC4299606

[33] Hannigan GD, Duhaime MB, Ruffin M t., Koumpouras CC, Schloss PD. 2018. Diagnostic potential and interactive dynamics of the colorectal cancer virome. mBio 9:e02248-18. doi:10.1128/mBio.02248-18 30459201 PMC6247079

[34] Yu J, Feng Q, Wong SH, Zhang D, Liang Q yi, Qin Y, Tang L, Zhao H, Stenvang J, Li Y, et al.. 2017. Metagenomic analysis of faecal microbiome as a tool towards targeted non-invasive biomarkers for colorectal cancer. Gut 66:70–78. doi:10.1136/gutjnl-2015-309800 26408641

[35] Liu N-N, Jiao N, Tan J-C, Wang Z, Wu D, Wang A-J, Chen J, Tao L, Zhou C, Fang W, Cheong IH, Pan W, Liao W, Kozlakidis Z, Heeschen C, Moore GG, Zhu L, Chen X, Zhang G, Zhu R, Wang H. 2022. Multi-kingdom microbiota analyses identify bacterial-fungal interactions and biomarkers of colorectal cancer across cohorts. Nat Microbiol 7:238–250. doi:10.1038/s41564-021-01030-7 35087227 PMC8813618

[36] Kielkowski P, Buchsbaum IY, Kirsch VC, Bach NC, Drukker M, Cappello S, Sieber SA. 2020. FICD activity and AMPylation remodelling modulate human neurogenesis. Nat Commun 11:517. doi:10.1038/s41467-019-14235-6 31980631 PMC6981130

[37] Fauser J, Gulen B, Pogenberg V, Pett C, Pourjafar-Dehkordi D, Krisp C, Höpfner D, König G, Schlüter H, Feige MJ, Zacharias M, Hedberg C, Itzen A. 2021. Specificity of AMPylation of the human chaperone BiP is mediated by TPR motifs of FICD. Nat Commun 12:2426. doi:10.1038/s41467-021-22596-0 33893288 PMC8065156

[38] Gulen B, Itzen A. 2022. Revisiting AMPylation through the lens of Fic enzymes. Trends Microbiol 30:350–363. doi:10.1016/j.tim.2021.08.003 34531089

[39] Mirdita M, Schütze K, Moriwaki Y, Heo L, Ovchinnikov S, Steinegger M. 2022. ColabFold: making protein folding accessible to all. Nat Methods 19:679–682. doi:10.1038/s41592-022-01488-1 35637307 PMC9184281

[40] Jumper J, Evans R, Pritzel A, Green T, Figurnov M, Ronneberger O, Tunyasuvunakool K, Bates R, Žídek A, Potapenko A, et al.. 2021. Highly accurate protein structure prediction with AlphaFold. Nature New Biol 596:583–589. doi:10.1038/s41586-021-03819-2 PMC8371605 34265844

[41] Strauss J, Kaplan GG, Beck PL, Rioux K, Panaccione R, Devinney R, Lynch T, Allen-Vercoe E. 2011. Invasive potential of gut mucosa-derived Fusobacterium nucleatum positively correlates with IBD status of the host. Inflamm Bowel Dis 17:1971–1978. doi:10.1002/ibd.21606 21830275

[42] Brennan CA, Clay SL, Lavoie SL, Bae S, Lang JK, Fonseca-Pereira D, Rosinski KG, Ou N, Glickman JN, Garrett WS. 2021. Fusobacterium nucleatum drives a pro-inflammatory intestinal microenvironment through metabolite receptor-dependent modulation of IL-17 expression. Gut Microbes 13:1987780. doi:10.1080/19490976.2021.1987780 34781821 PMC8604392

[43] Manson McGuire A, Cochrane K, Griggs AD, Haas BJ, Abeel T, Zeng Q, Nice JB, MacDonald H, Birren BW, Berger BW, Allen-Vercoe E, Earl AM. 2014. Evolution of invasion in a diverse set of Fusobacterium species. mBio 5:e01864-14. doi:10.1128/mBio.01864-14 25370491 PMC4222103

[44] Flynn JM, Hubley R, Goubert C, Rosen J, Clark AG, Feschotte C, Smit AF. 2020. RepeatModeler2 for automated genomic discovery of transposable element families. Proc Natl Acad Sci U S A 117:9451–9457. doi:10.1073/pnas.1921046117 32300014 PMC7196820

[45] Chevez-Guardado R, Peña-Castillo L. 2021. Promotech: a general tool for bacterial promoter recognition. Genome Biol 22:318. doi:10.1186/s13059-021-02514-9 34789306 PMC8597233

[46] Steinegger M, Söding J. 2017. MMseqs2 enables sensitive protein sequence searching for the analysis of massive data sets. Nat Biotechnol 35:1026–1028. doi:10.1038/nbt.3988 29035372

[47] Corbett TH, Griswold DP, Roberts BJ, Peckham JC, Schabel FM Jr. 1975. Tumor induction relationships in development of transplantable cancers of the colon in mice for chemotherapy assays, with a note on carcinogen structure. Cancer Res 35:2434–2439. 1149045

[48] Sato Y, Fu Y, Liu H, Lee MY, Shaw MH. 2021. Tumor-immune profiling of CT-26 and Colon 26 syngeneic mouse models reveals mechanism of anti-PD-1 response. BMC Cancer 21:1222. doi:10.1186/s12885-021-08974-3 34774008 PMC8590766

[49] Murphy RJ, Gunasingh G, Haass NK, Simpson MJ. 2023. Growth and adaptation mechanisms of tumour spheroids with time-dependent oxygen availability. PLoS Comput Biol 19:e1010833. doi:10.1371/journal.pcbi.1010833 36634128 PMC9876349

[50] Godet I, Shin YJ, Ju JA, Ye IC, Wang G, Gilkes DM. 2019. Fate-mapping post-hypoxic tumor cells reveals a ROS-resistant phenotype that promotes metastasis. Nat Commun 10:4862. doi:10.1038/s41467-019-12412-1 31649238 PMC6813355

[51] Brennan CA, Nakatsu G, Gallini Comeau CA, Drew DA, Glickman JN, Schoen RE, Chan AT, Garrett WS. 2021. Aspirin modulation of the colorectal cancer-associated microbe Fusobacterium nucleatum. mBio 12:e00547-21. doi:10.1128/mBio.00547-21 33824205 PMC8092249

[52] Doron L, Coppenhagen-Glazer S, Ibrahim Y, Eini A, Naor R, Rosen G, Bachrach G. 2014. Identification and characterization of fusolisin, the Fusobacterium nucleatum autotransporter serine protease. PLoS One 9:e111329. doi:10.1371/journal.pone.0111329 25357190 PMC4214739

[53] Matsuda S, Okada R, Tandhavanant S, Hiyoshi H, Gotoh K, Iida T, Kodama T. 2019. Export of a Vibrio parahaemolyticus toxin by the Sec and type III secretion machineries in tandem. Nat Microbiol 4:781–788. doi:10.1038/s41564-019-0368-y 30778145

[54] Fromm K, Dehio C. 2021. The impact of Bartonella VirB/VirD4 type IV secretion system effectors on eukaryotic host cells. Front Microbiol 12:762582. doi:10.3389/fmicb.2021.762582 34975788 PMC8714903

[55] Meir A, Macé K, Lukoyanova N, Chetrit D, Hospenthal MK, Redzej A, Roy C, Waksman G. 2020. Mechanism of effector capture and delivery by the type IV secretion system from Legionella pneumophila. Nat Commun 11:2864. doi:10.1038/s41467-020-16681-z 32513920 PMC7280309

[56] Higa N, Toma C, Koizumi Y, Nakasone N, Nohara T, Masumoto J, Kodama T, Iida T, Suzuki T. 2013. Vibrio parahaemolyticus effector proteins suppress inflammasome activation by interfering with host autophagy signaling. PLoS Pathog 9:e1003142. doi:10.1371/journal.ppat.1003142 23357873 PMC3554609

[57] Wu Y, Guo S, Chen F, Li Y, Huang Y, Liu W, Zhang G. 2023. Fn-Dps, a novel virulence factor of Fusobacterium nucleatum, disrupts erythrocytes and promotes metastasis in colorectal cancer. PLoS Pathog 19:e1011096. doi:10.1371/journal.ppat.1011096 36693067 PMC9873182

[58] Pastushenko I, Blanpain C. 2019. EMT transition states during tumor progression and metastasis. Trends Cell Biol 29:212–226. doi:10.1016/j.tcb.2018.12.001 30594349

[59] Zhang N, Ng AS, Cai S, Li Q, Yang L, Kerr D. 2021. Novel therapeutic strategies: targeting epithelial-mesenchymal transition in colorectal cancer. Lancet Oncol 22:e358–e368. doi:10.1016/S1470-2045(21)00343-0 34339656

[60] Veyron S, Oliva G, Rolando M, Buchrieser C, Peyroche G, Cherfils J. 2019. A Ca^2+^-regulated deAMPylation switch in human and bacterial FIC proteins. Nat Commun 10:1142. doi:10.1038/s41467-019-09023-1 30850593 PMC6408439

[61] Dedic E, Alsarraf H, Welner DH, Østergaard O, Klychnikov OI, Hensbergen PJ, Corver J, van Leeuwen HC, Jørgensen R. 2016. A novel fic (filamentation induced by cAMP) protein from Clostridium difficile reveals an inhibitory motif-independent adenylylation/AMPylation mechanism. J Biol Chem 291:13286–13300. doi:10.1074/jbc.M115.705491 27076635 PMC4933240

[62] Boonanantanasarn K, Gill AL, Yap Y, Jayaprakash V, Sullivan MA, Gill SR. 2012. Enterococcus faecalis enhances cell proliferation through hydrogen peroxide-mediated epidermal growth factor receptor activation. Infect Immun 80:3545–3558. doi:10.1128/IAI.00479-12 22851748 PMC3457582

[63] Segelle A, Núñez-Álvarez Y, Oldfield AJ, Webb KM, Voigt P, Luco RF. 2022. Histone marks regulate the epithelial-to-mesenchymal transition via alternative splicing. Cell Rep 38:110357. doi:10.1016/j.celrep.2022.110357 35172149

[64] Serrano-Gomez SJ, Maziveyi M, Alahari SK. 2016. Regulation of epithelial-mesenchymal transition through epigenetic and post-translational modifications. Mol Cancer 15:18. doi:10.1186/s12943-016-0502-x 26905733 PMC4765192

[65] Höpfner D, Cichy A, Pogenberg V, Krisp C, Mezouar S, Bach NC, Grotheer J, Zarza SM, Martinez E, Bonazzi M, Feige MJ, Sieber SA, Schlüter H, Itzen A. 2023. The DNA-binding induced (de)AMPylation activity of a Coxiella burnetii Fic enzyme targets Histone H3. Commun Biol 6:1124. doi:10.1038/s42003-023-05494-7 37932372 PMC10628234

[66] Wright BW, Molloy MP, Jaschke PR. 2022. Overlapping genes in natural and engineered genomes. Nat Rev Genet 23:154–168. doi:10.1038/s41576-021-00417-w 34611352 PMC8490965

[67] Wilde J, Allen-Vercoe E. 2023. Characterizing prophages in the genus Fusobacterium. Anaerobe 80:102718. doi:10.1016/j.anaerobe.2023.102718 36801248

[68] Cochrane K, McGuire AM, Priest ME, Abouelleil A, Cerqueira GC, Lo R, Earl AM, Allen-Vercoe E. 2016. Complete genome sequences and analysis of the Fusobacterium nucleatum subspecies animalis 7-1 bacteriophage ɸFunu1 and ɸFunu2. Anaerobe 38:125–129. doi:10.1016/j.anaerobe.2015.10.013 26545740 PMC4775352

[69] Fremin BJ, Bhatt AS, Kyrpides NC, Global Phage Small Open Reading Frame C. 2022. Thousands of small, novel genes predicted in global phage genomes. Cell Rep 39:110984. doi:10.1016/j.celrep.2022.110984 35732113 PMC9254267

[70] Silpe JE, Duddy OP, Johnson GE, Beggs GA, Hussain FA, Forsberg KJ, Bassler BL. 2023. Small protein modules dictate prophage fates during polylysogeny. Nature New Biol 620:625–633. doi:10.1038/s41586-023-06376-y PMC10432266 37495698

[71] Burns N, James CE, Harrison E. 2015. Polylysogeny magnifies competitiveness of a bacterial pathogen in vivo. Evol Appl 8:346–351. doi:10.1111/eva.12243 25926879 PMC4408145

[72] Bohlin J, Eldholm V, Pettersson JHO, Brynildsrud O, Snipen L. 2017. The nucleotide composition of microbial genomes indicates differential patterns of selection on core and accessory genomes. BMC Genomics 18:151. doi:10.1186/s12864-017-3543-7 28187704 PMC5303225

[73] Bohlin J, Snipen L, Hardy SP, Kristoffersen AB, Lagesen K, Dønsvik T, Skjerve E, Ussery DW. 2010. Analysis of intra-genomic GC content homogeneity within prokaryotes. BMC Genomics 11:464. doi:10.1186/1471-2164-11-464 20691090 PMC3091660

[74] Abed J, Maalouf N, Manson AL, Earl AM, Parhi L, Emgård JEM, Klutstein M, Tayeb S, Almogy G, Atlan KA, Chaushu S, Israeli E, Mandelboim O, Garrett WS, Bachrach G. 2020. Colon cancer-associated Fusobacterium nucleatum may originate from the oral cavity and reach colon tumors via the circulatory system. Front Cell Infect Microbiol 10:400. doi:10.3389/fcimb.2020.00400 32850497 PMC7426652

[75] Despins CA, Brown SD, Robinson AV, Mungall AJ, Allen-Vercoe E, Holt RA. 2021. Modulation of the host cell transcriptome and epigenome by Fusobacterium nucleatum. mBio 12:e02062-21. doi:10.1128/mBio.02062-21 34700376 PMC8546542

[76] El Tekle G, Garrett WS. 2023. Bacteria in cancer initiation, promotion and progression. Nat Rev Cancer 23:600–618. doi:10.1038/s41568-023-00594-2 37400581

[77] Brennan CA, Garrett WS. 2019. Fusobacterium nucleatum - symbiont, opportunist and oncobacterium. Nat Rev Microbiol 17:156–166. doi:10.1038/s41579-018-0129-6 30546113 PMC6589823

[78] El Tekle G, Andreeva N, Garrett WS. 2024. The role of the microbiome in the etiopathogenesis of colon cancer. Annu Rev Physiol 86:453–478. doi:10.1146/annurev-physiol-042022-025619 38345904

[79] Clay SL, Fonseca-Pereira D, Garrett WS. 2022. Colorectal cancer: the facts in the case of the microbiota. J Clin Invest 132:e155101. doi:10.1172/JCI155101 35166235 PMC8843708

[80] Zhang L, Leng X-X, Qi J, Wang N, Han J-X, Tao Z-H, Zhuang Z-Y, Ren Y, Xie Y-L, Jiang S-S, Li J-L, Chen H, Zhou C-B, Cui Y, Chen X, Wang Z, Zhang Z-Z, Hong J, Chen H-Y, Jiang W, Chen Y-X, Zhao X, Yu J, Fang J-Y. 2024. The adhesin RadD enhances Fusobacterium nucleatum tumour colonization and colorectal carcinogenesis. Nat Microbiol 9:2292–2307. doi:10.1038/s41564-024-01784-w 39169124

[81] Hanahan D. 2022. Hallmarks of cancer: new dimensions. Cancer Discov 12:31–46. doi:10.1158/2159-8290.CD-21-1059 35022204

[82] Almeida A, Nayfach S, Boland M, Strozzi F, Beracochea M, Shi ZJ, Pollard KS, Sakharova E, Parks DH, Hugenholtz P, Segata N, Kyrpides NC, Finn RD. 2021. A unified catalog of 204,938 reference genomes from the human gut microbiome. Nat Biotechnol 39:105–114. doi:10.1038/s41587-020-0603-3 32690973 PMC7801254

[83] Kieser S, Zdobnov EM, Trajkovski M. 2022. Comprehensive mouse microbiota genome catalog reveals major difference to its human counterpart. PLoS Comput Biol 18:e1009947. doi:10.1371/journal.pcbi.1009947 35259160 PMC8932566

[84] Leviatan S, Shoer S, Rothschild D, Gorodetski M, Segal E. 2022. An expanded reference map of the human gut microbiome reveals hundreds of previously unknown species. Nat Commun 13:3863. doi:10.1038/s41467-022-31502-1 35790781 PMC9256738

[85] Lin X, Hu T, Chen J, Liang H, Zhou J, Wu Z, Ye C, Jin X, Xu X, Zhang W, Jing X, Yang T, Wang J, Yang H, Kristiansen K, Xiao L, Zou Y. 2023. The genomic landscape of reference genomes of cultivated human gut bacteria. Nat Commun 14:1663. doi:10.1038/s41467-023-37396-x 36966151 PMC10039858

[86] Poyet M, Groussin M, Gibbons SM, Avila-Pacheco J, Jiang X, Kearney SM, Perrotta AR, Berdy B, Zhao S, Lieberman TD, Swanson PK, Smith M, Roesemann S, Alexander JE, Rich SA, Livny J, Vlamakis H, Clish C, Bullock K, Deik A, Scott J, Pierce KA, Xavier RJ, Alm EJ. 2019. A library of human gut bacterial isolates paired with longitudinal multiomics data enables mechanistic microbiome research. Nat Med 25:1442–1452. doi:10.1038/s41591-019-0559-3 31477907

[87] Wu L, Ma J. 2019. The global catalogue of microorganisms (GCM) 10K type strain sequencing project: providing services to taxonomists for standard genome sequencing and annotation. Int J Syst Evol Microbiol 69:895–898. doi:10.1099/ijsem.0.003276 30832757

[88] Zou Y, Xue W, Luo G, Deng Z, Qin P, Guo R, Sun H, Xia Y, Liang S, Dai Y, et al.. 2019. 1,520 reference genomes from cultivated human gut bacteria enable functional microbiome analyses. Nat Biotechnol 37:179–185. doi:10.1038/s41587-018-0008-8 30718868 PMC6784896

[89] Sears CL. 2009. Enterotoxigenic Bacteroides fragilis: a rogue among symbiotes. Clin Microbiol Rev 22:349–369, doi:10.1128/CMR.00053-08 19366918 PMC2668231

[90] Chen B, Ramazzotti D, Heide T, Spiteri I, Fernandez-Mateos J, James C, Magnani L, Graham TA, Sottoriva A. 2023. Contribution of pks+ E. coli mutations to colorectal carcinogenesis. Nat Commun 14:7827. doi:10.1038/s41467-023-43329-5 38030613 PMC10687070

[91] Pleguezuelos-Manzano C, Puschhof J, Rosendahl Huber A, van Hoeck A, Wood HM, Nomburg J, Gurjao C, Manders F, Dalmasso G, Stege PB, et al.. 2020. Mutational signature in colorectal cancer caused by genotoxic pks+ E. coli. Nature New Biol 580:269–273. doi:10.1038/s41586-020-2080-8 PMC8142898 32106218

[92] Arima K, Zhong R, Ugai T, Zhao M, Haruki K, Akimoto N, Lau MC, Okadome K, Mehta RS, Väyrynen JP, et al.. 2022. Western-style diet, pks Island-carrying Escherichia coli, and colorectal cancer: analyses from Two large prospective cohort studies. Gastroenterology 163:862–874. doi:10.1053/j.gastro.2022.06.054 35760086 PMC9509428

[93] Pedruzzi I, Rivoire C, Auchincloss AH, Coudert E, Keller G, de Castro E, Baratin D, Cuche BA, Bougueleret L, Poux S, Redaschi N, Xenarios I, Bridge A. 2015. HAMAP in 2015: updates to the protein family classification and annotation system. Nucleic Acids Res 43:D1064–70. doi:10.1093/nar/gku1002 25348399 PMC4383873

[94] Mistry J, Chuguransky S, Williams L, Qureshi M, Salazar GA, Sonnhammer ELL, Tosatto SCE, Paladin L, Raj S, Richardson LJ, Finn RD, Bateman A. 2021. Pfam: the protein families database in 2021. Nucleic Acids Res 49:D412–D419. doi:10.1093/nar/gkaa913 33125078 PMC7779014

[95] Chaumeil PA, Mussig AJ, Hugenholtz P, Parks DH. 2019. GTDB-Tk: a toolkit to classify genomes with the genome taxonomy database. Bioinformatics 36:1925–1927. doi:10.1093/bioinformatics/btz848 31730192 PMC7703759

[96] Parks DH, Chuvochina M, Waite DW, Rinke C, Skarshewski A, Chaumeil P-A, Hugenholtz P. 2018. A standardized bacterial taxonomy based on genome phylogeny substantially revises the tree of life. Nat Biotechnol 36:996–1004. doi:10.1038/nbt.4229 30148503

[97] Ruscheweyh H-J, Milanese A, Paoli L, Karcher N, Clayssen Q, Keller MI, Wirbel J, Bork P, Mende DR, Zeller G, Sunagawa S. 2022. Cultivation-independent genomes greatly expand taxonomic-profiling capabilities of mOTUs across various environments. Microbiome 10:212. doi:10.1186/s40168-022-01410-z 36464731 PMC9721005

[98] Bolger AM, Lohse M, Usadel B. 2014. Trimmomatic: a flexible trimmer for Illumina sequence data. Bioinformatics 30:2114–2120. doi:10.1093/bioinformatics/btu170 24695404 PMC4103590

[99] Li H., Durbin R. 2009. Fast and accurate short read alignment with Burrows-Wheeler transform. Bioinformatics 25:1754–1760. doi:10.1093/bioinformatics/btp324 19451168 PMC2705234

[100] McKenna A, Hanna M, Banks E, Sivachenko A, Cibulskis K, Kernytsky A, Garimella K, Altshuler D, Gabriel S, Daly M, DePristo MA. 2010. The genome analysis Toolkit: a MapReduce framework for analyzing next-generation DNA sequencing data. Genome Res 20:1297–1303. doi:10.1101/gr.107524.110 20644199 PMC2928508

[101] Li H, Handsaker B, Wysoker A, Fennell T, Ruan J, Homer N, Marth G, Abecasis G, Durbin R, 1000 Genome Project Data Processing Subgroup. 2009. The sequence alignment/Map format and SAMtools. Bioinformatics 25:2078–2079. doi:10.1093/bioinformatics/btp352 19505943 PMC2723002

[102] Nurk S, Koren S, Rhie A, Rautiainen M, Bzikadze AV, Mikheenko A, Vollger MR, Altemose N, Uralsky L, Gershman A, et al.. 2022. The complete sequence of a human genome. Science 376:44–53. doi:10.1126/science.abj6987 35357919 PMC9186530

[103] Nayfach S, Pollard KS. 2015. Average genome size estimation improves comparative metagenomics and sheds light on the functional ecology of the human microbiome. Genome Biol 16:51. doi:10.1186/s13059-015-0611-7 25853934 PMC4389708

[104] Levin BJ, Huang YY, Peck SC, Wei Y, Martínez-Del Campo A, Marks JA, Franzosa EA, Huttenhower C, Balskus EP. 2017. A prominent glycyl radical enzyme in human gut microbiomes metabolizes trans-4-hydroxy-l-proline. Science 355:eaai8386. doi:10.1126/science.aai8386 28183913 PMC5705181

[105] Saenz C, Nigro E, Gunalan V, Arumugam M. 2022. MIntO: a modular and scalable pipeline for microbiome metagenomic and metatranscriptomic data integration. Front Bioinform 2:846922. doi:10.3389/fbinf.2022.846922 36304282 PMC9580859

[106] Gautreau G, Bazin A, Gachet M, Planel R, Burlot L, Dubois M, Perrin A, Médigue C, Calteau A, Cruveiller S, Matias C, Ambroise C, Rocha EPC, Vallenet D. 2020. PPanGGOLiN: depicting microbial diversity via a partitioned pangenome graph. PLoS Comput Biol 16:e1007732. doi:10.1371/journal.pcbi.1007732 32191703 PMC7108747

[107] Paradis E, Schliep K. 2019. Ape 5.0: an environment for modern phylogenetics and evolutionary analyses in R. Bioinformatics 35:526–528. doi:10.1093/bioinformatics/bty633 30016406

[108] Yu G. 2020. Using ggtree to visualize data on tree-like structures. Curr Protoc Bioinformatics 69:e96. doi:10.1002/cpbi.96 32162851

[109] Jacomy M, Venturini T, Heymann S, Bastian M. 2014. ForceAtlas2, a continuous graph layout algorithm for handy network visualization designed for the Gephi software. PLoS One 9:e98679. doi:10.1371/journal.pone.0098679 24914678 PMC4051631

[110] Addou S, Rentzsch R, Lee D, Orengo CA. 2009. Domain-based and family-specific sequence identity thresholds increase the levels of reliable protein function transfer. J Mol Biol 387:416–430. doi:10.1016/j.jmb.2008.12.045 19135455

[111] Sangar V, Blankenberg DJ, Altman N, Lesk AM. 2007. Quantitative sequence-function relationships in proteins based on gene ontology. BMC Bioinformatics 8:294. doi:10.1186/1471-2105-8-294 17686158 PMC1976327

[112] Bodenhofer U, Bonatesta E, Horejš-Kainrath C, Hochreiter S. 2015. Msa: an R package for multiple sequence alignment. Bioinformatics 31:3997–3999. doi:10.1093/bioinformatics/btv494 26315911

[113] Zhou L, Feng T, Xu S, Gao F, Lam TT, Wang Q, Wu T, Huang H, Zhan L, Li L, Guan Y, Dai Z, Yu G. 2022. Ggmsa: a visual exploration tool for multiple sequence alignment and associated data. Brief Bioinform 23:bbac222. doi:10.1093/bib/bbac222 35671504

[114] Wagih O. 2017. Ggseqlogo: a versatile R package for drawing sequence logos. Bioinformatics 33:3645–3647. doi:10.1093/bioinformatics/btx469 29036507

[115] Quinlan AR, Hall IM. 2010. BEDTools: a flexible suite of utilities for comparing genomic features. Bioinformatics 26:841–842. doi:10.1093/bioinformatics/btq033 20110278 PMC2832824

[116] Li H. 2018. Minimap2: pairwise alignment for nucleotide sequences. Bioinformatics 34:3094–3100. doi:10.1093/bioinformatics/bty191 29750242 PMC6137996

[117] Shen W, Le S, Li Y, Hu F. 2016. SeqKit: a cross-platform and ultrafast toolkit for FASTA/Q file manipulation. PLoS ONE 11:e0163962. doi:10.1371/journal.pone.0163962 27706213 PMC5051824

[118] Bruggeling CE, Garza DR, Achouiti S, Mes W, Dutilh BE, Boleij A. 2021. Optimized bacterial DNA isolation method for microbiome analysis of human tissues. Microbiologyopen 10:e1191. doi:10.1002/mbo3.1191 34180607 PMC8208965

[119] Smolander N, Julian TR, Tamminen M. 2022. Prider: multiplexed primer design using linearly scaling approximation of set coverage. BMC Bioinformatics 23:174. doi:10.1186/s12859-022-04710-1 35549665 PMC9097127

[120] Ye J, Coulouris G, Zaretskaya I, Cutcutache I, Rozen S, Madden TL. 2012. Primer-BLAST: a tool to design target-specific primers for polymerase chain reaction. BMC Bioinformatics 13:134. doi:10.1186/1471-2105-13-134 22708584 PMC3412702

[121] Vandesompele J, De Preter K, Pattyn F, Poppe B, Van Roy N, De Paepe A, Speleman F. 2002. Accurate normalization of real-time quantitative RT-PCR data by geometric averaging of multiple internal control genes. Genome Biol 3:RESEARCH0034. doi:10.1186/gb-2002-3-7-research0034 12184808 PMC126239

[122] Dembélé D, Kastner P. 2014. Fold change rank ordering statistics: a new method for detecting differentially expressed genes. BMC Bioinformatics 15:14. doi:10.1186/1471-2105-15-14 24423217 PMC3899927

[123] Hulstaert N, Shofstahl J, Sachsenberg T, Walzer M, Barsnes H, Martens L, Perez-Riverol Y. 2020. ThermoRawFileParser: modular, scalable, and cross-platform RAW file conversion. J Proteome Res 19:537–542. doi:10.1021/acs.jproteome.9b00328 31755270 PMC7116465

[124] Kim S, Pevzner PA. 2014. MS-GF+ makes progress towards a universal database search tool for proteomics. Nat Commun 5:5277. doi:10.1038/ncomms6277 25358478 PMC5036525

[125] Song Y, Wang J. 2023. Ggcoverage: an R package to visualize and annotate genome coverage for various NGS data. BMC Bioinformatics 24:309. doi:10.1186/s12859-023-05438-2 37559015 PMC10413535

[126] Bao K, Bostanci N, Thurnheer T, Grossmann J, Wolski WE, Thay B, Belibasakis GN, Oscarsson J. 2018. Aggregatibacter actinomycetemcomitans H-NS promotes biofilm formation and alters protein dynamics of other species within a polymicrobial oral biofilm. NPJ Biofilms Microbiomes 4:12. doi:10.1038/s41522-018-0055-4 29844920 PMC5964231

[127] Ali Mohammed MM, Pettersen VK, Nerland AH, Wiker HG, Bakken V. 2021. Label-free quantitative proteomic analysis of the oral bacteria Fusobacterium nucleatum and Porphyromonas gingivalis to identify protein features relevant in biofilm formation. Anaerobe 72:102449. doi:10.1016/j.anaerobe.2021.102449 34543761

[128] Mohammed MMA, Pettersen VK, Nerland AH, Wiker HG, Bakken V. 2017. Quantitative proteomic analysis of extracellular matrix extracted from mono- and dual-species biofilms of Fusobacterium nucleatum and Porphyromonas gingivalis. Anaerobe 44:133–142. doi:10.1016/j.anaerobe.2017.03.002 28285095

[129] Zhang X, Wang Y, Fan R, Zhang L, Li Z, Zhang Y, Zheng W, Wang L, Liu B, Quan C. 2023. Quantitative proteomic analysis of outer membrane vesicles from Fusobacterium nucleatum cultivated in the mimic cancer environment. Microbiol Spectr 11:e0039423. doi:10.1128/spectrum.00394-23 37341631 PMC10434195

[130] Vizcaíno JA, Deutsch EW, Wang R, Csordas A, Reisinger F, Ríos D, Dianes JA, Sun Z, Farrah T, Bandeira N, Binz P-A, Xenarios I, Eisenacher M, Mayer G, Gatto L, Campos A, Chalkley RJ, Kraus H-J, Albar JP, Martinez-Bartolomé S, Apweiler R, Omenn GS, Martens L, Jones AR, Hermjakob H. 2014. ProteomeXchange provides globally coordinated proteomics data submission and dissemination. Nat Biotechnol 32:223–226. doi:10.1038/nbt.2839 24727771 PMC3986813

