# Supplementary figures and images for: Virulence factor discovery identifies associations between the Fic gene family and Fap2+ fusobacteria in colorectal cancer microbiomes
Source: mBio. 2025 Jan 14;16(2):e03732-24. doi: 10.1128/mbio.03732-24 (PMC11796403; doi:10.1128/mbio.03732-24)

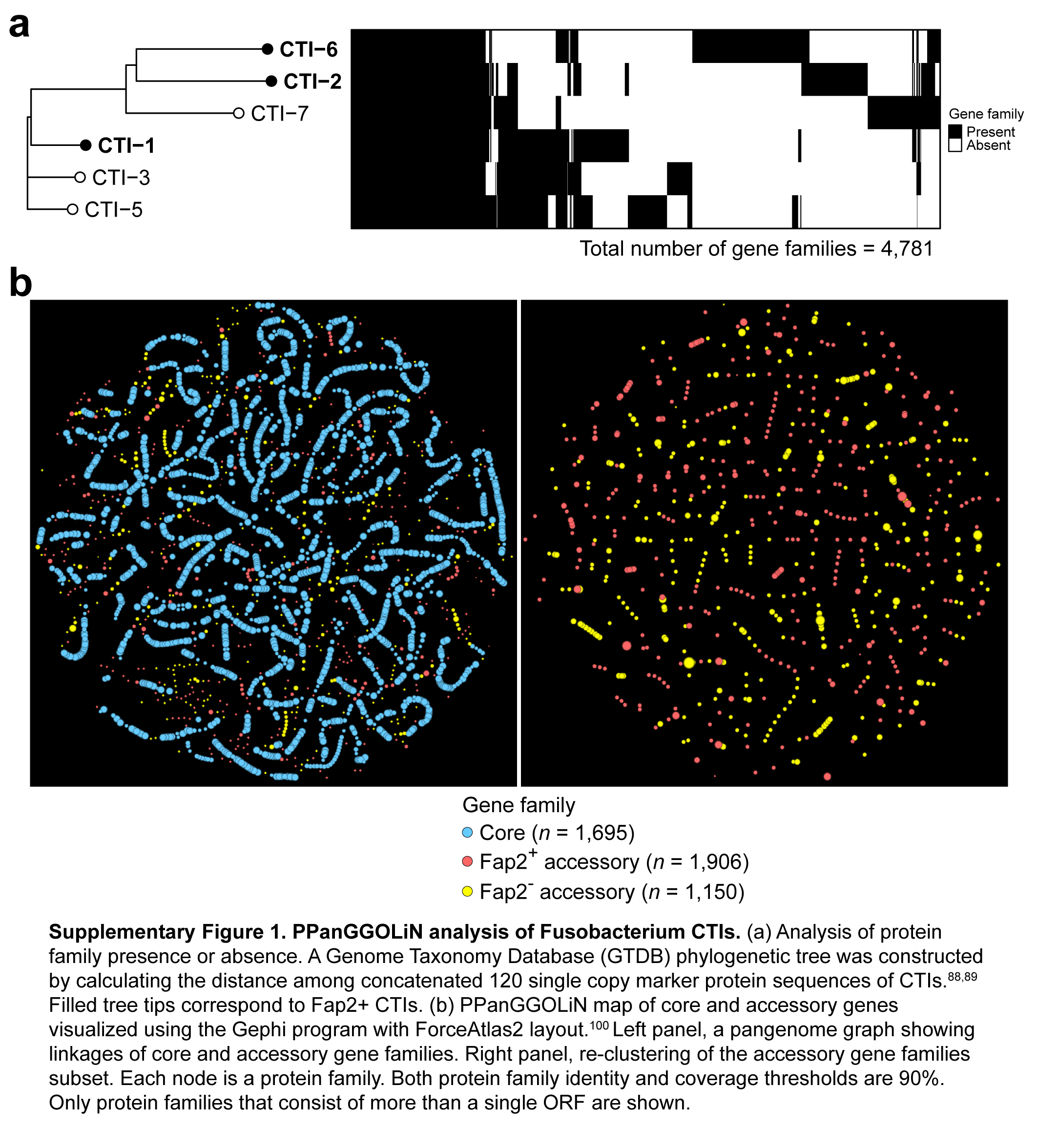

Supplement: Fig. S1 — PPanGGOLiN analysis of Fusobacterium CTIs. [file mbio.03732-24-s0001.tif]

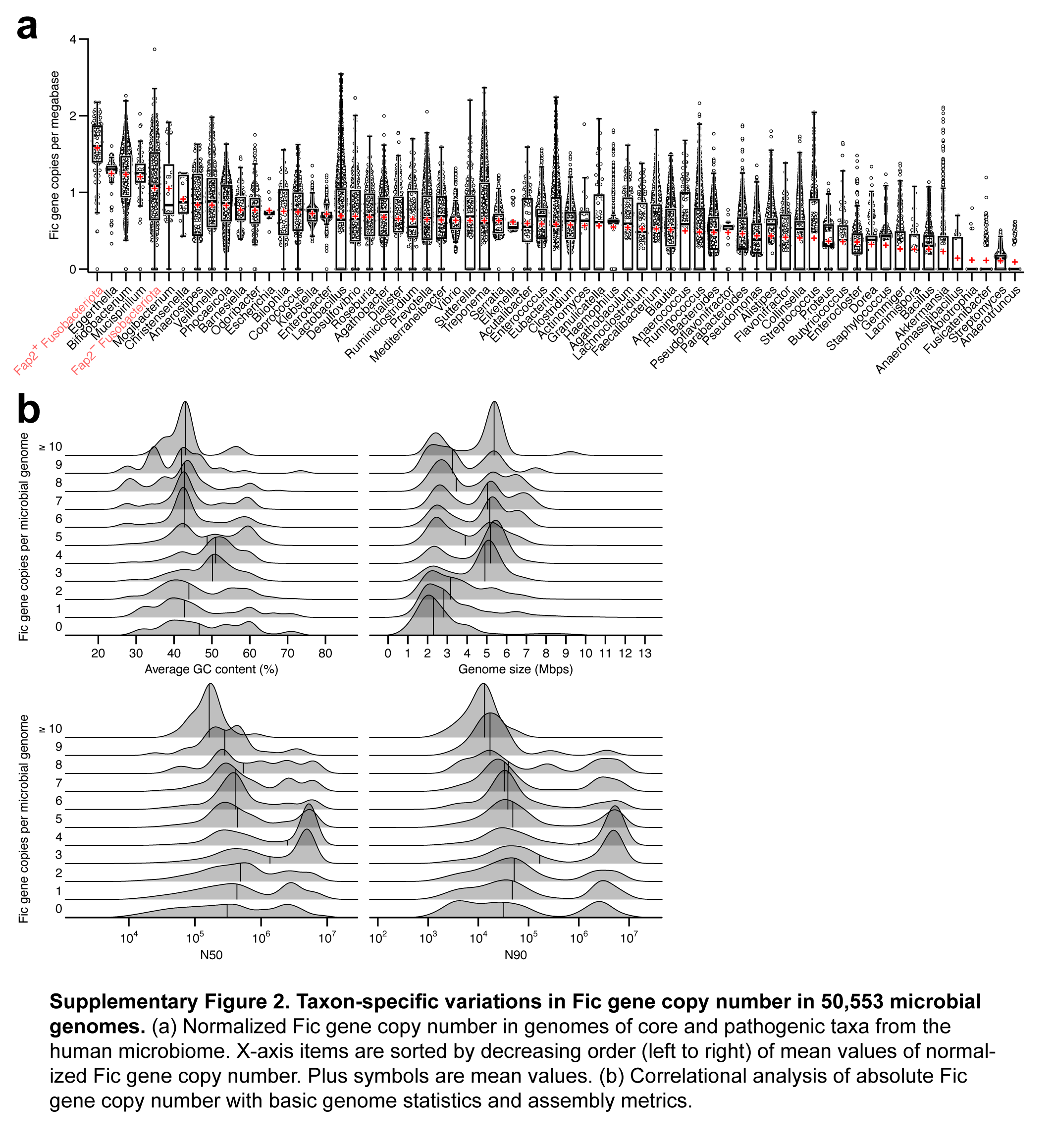

Supplement: Fig. S2 — Taxon-specific variations in Fic gene copy number in 50,553 microbial genomes. [file mbio.03732-24-s0002.tif]

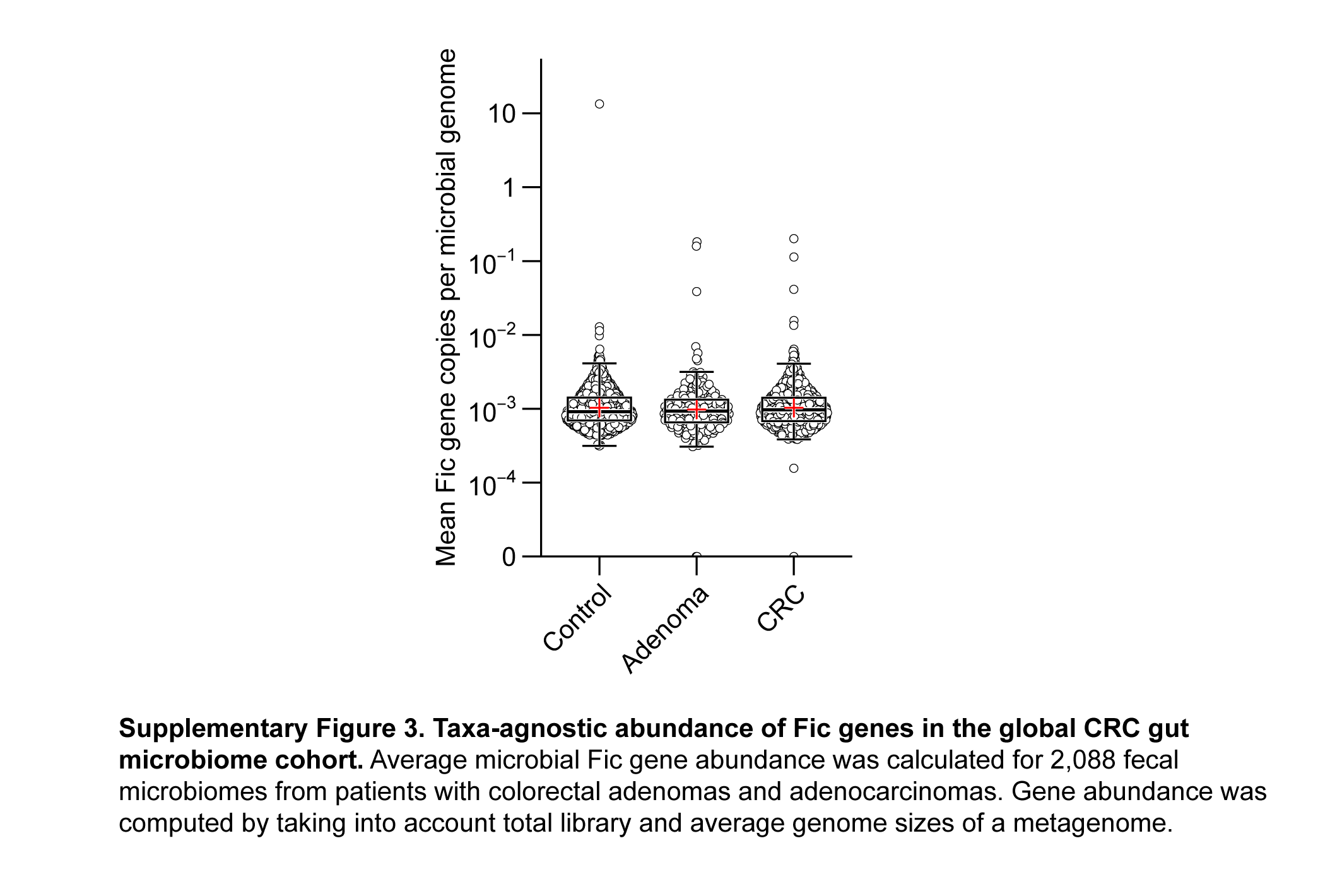

Supplement: Fig. S3 — Taxon-agnostic abundance of Fic genes in the global CRC gut microbiome cohort. [file mbio.03732-24-s0003.tif]

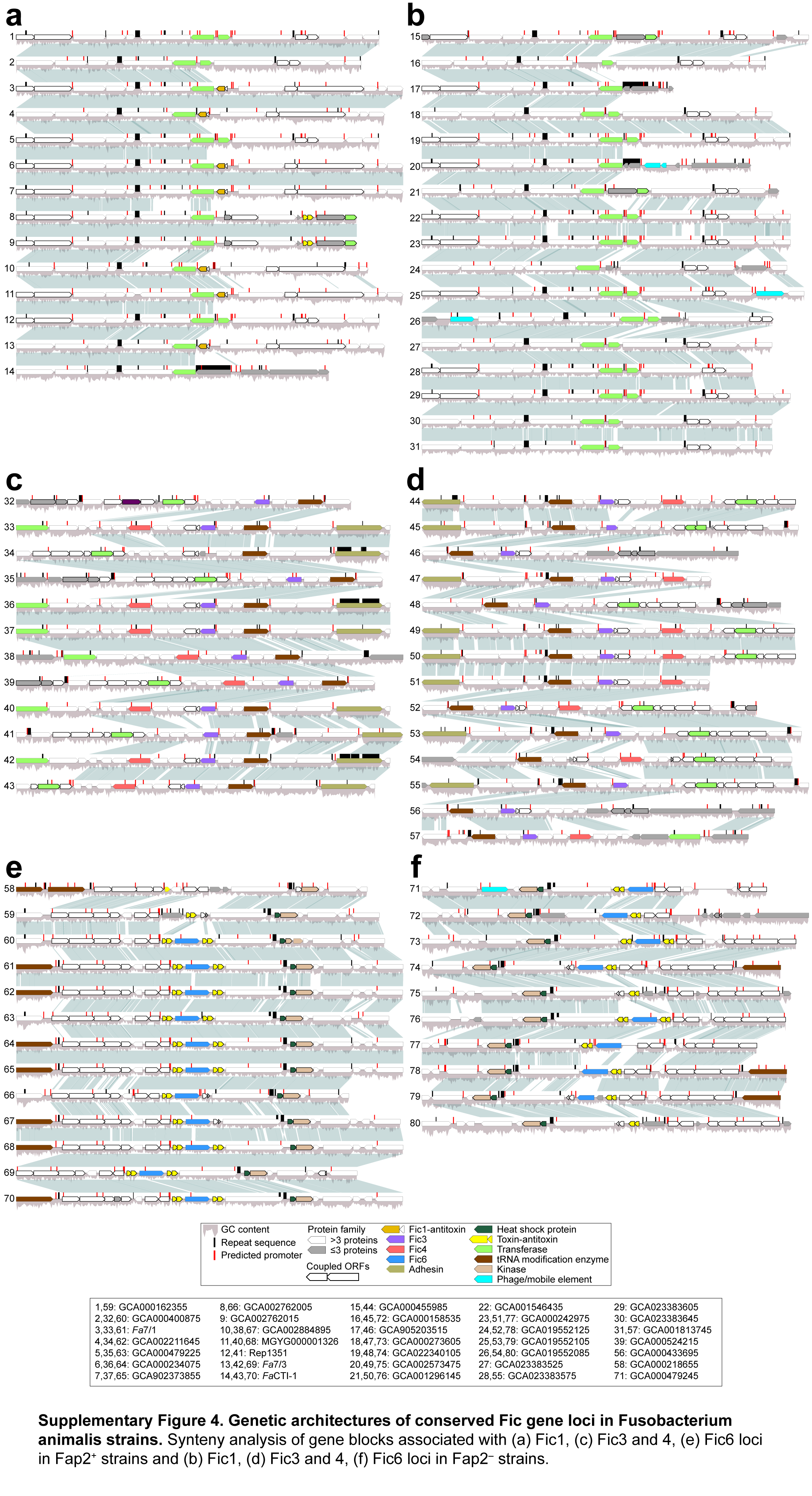

Supplement: Fig. S4 — Genetic architectures of conserved Fic gene loci in Fusobacterium animalis strains. [file mbio.03732-24-s0004.tif]

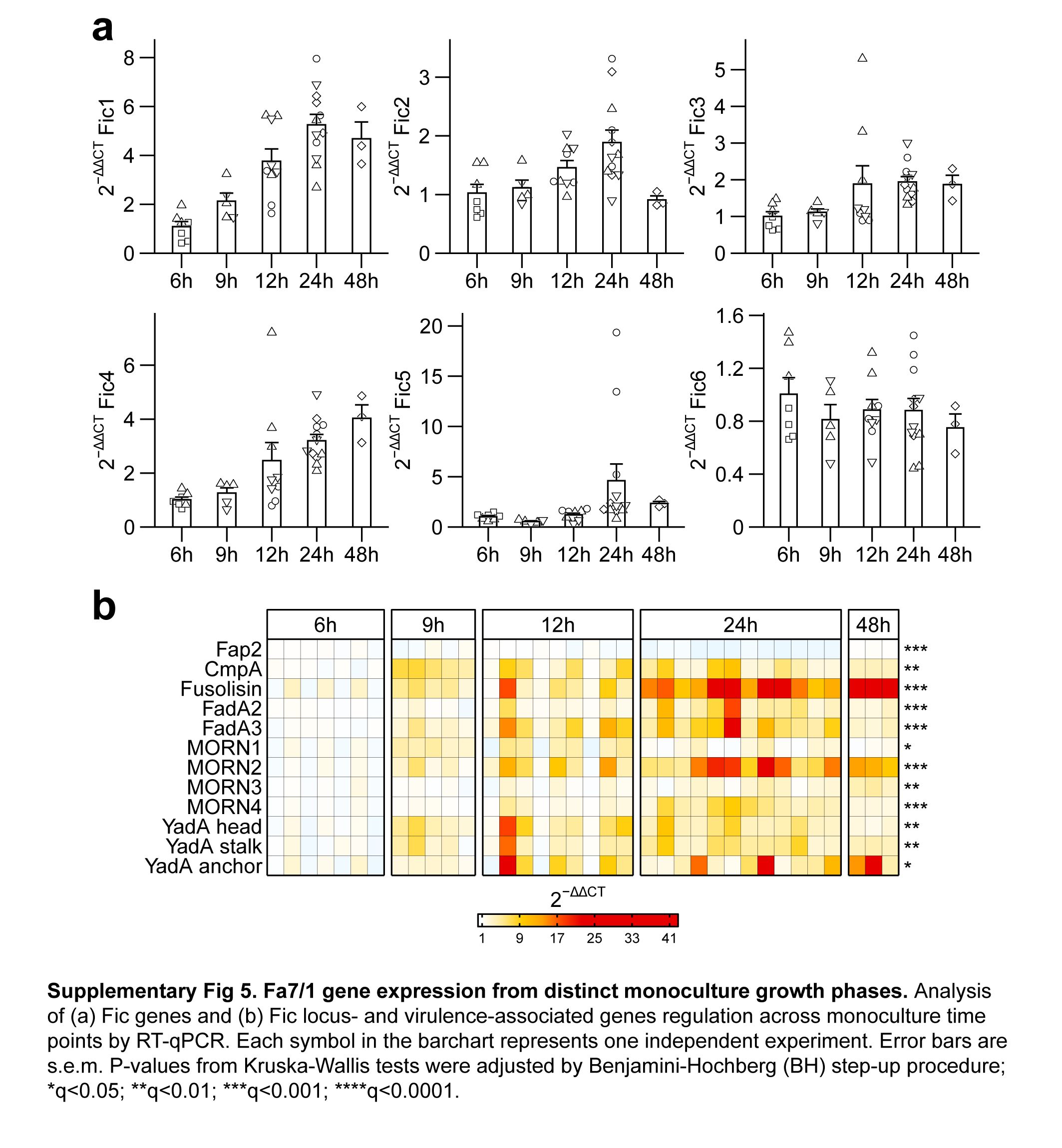

Supplement: Fig. S5 — Fa7/1 gene expression from distinct monoculture growth phases. [file mbio.03732-24-s0005.tif]

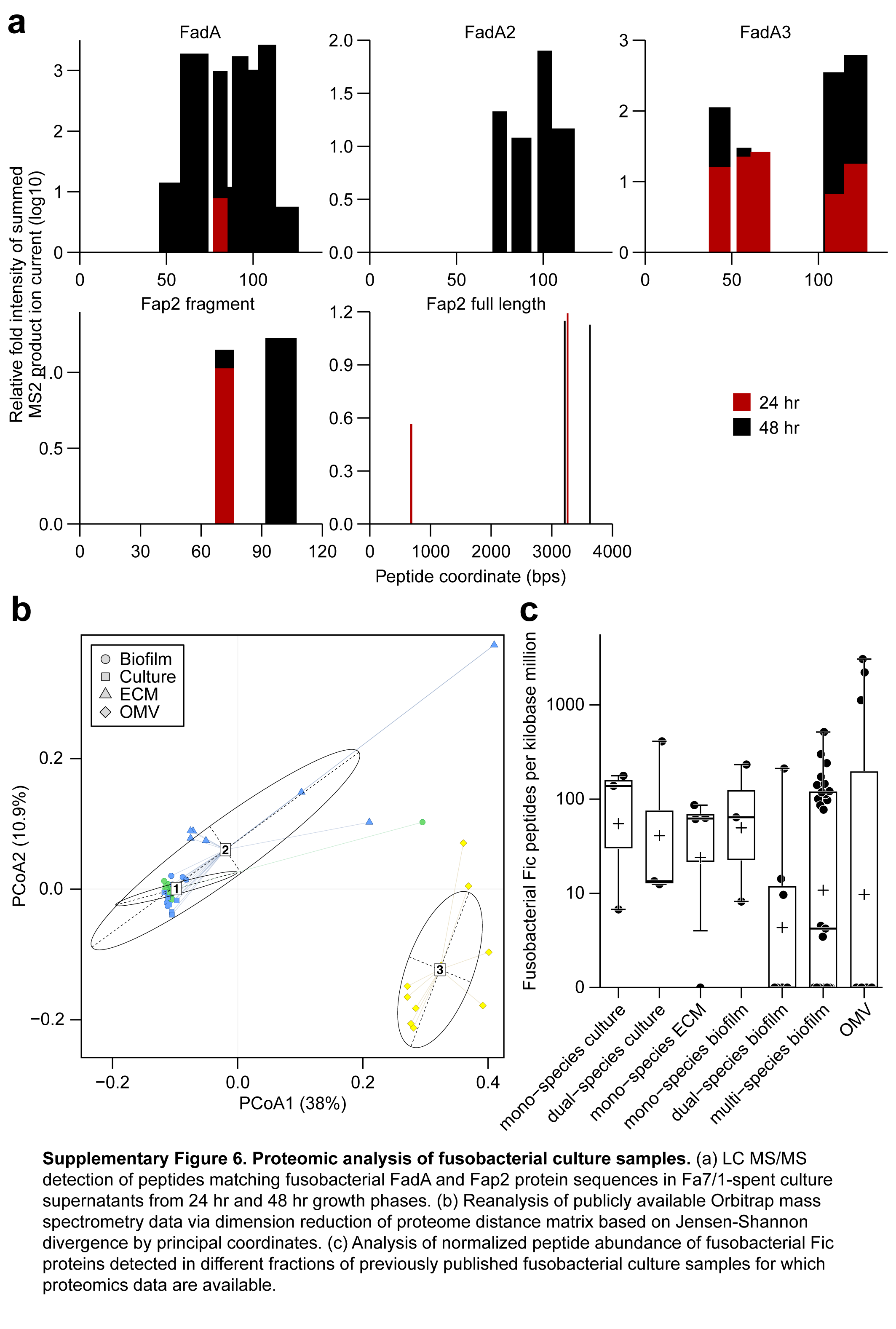

Supplement: Fig. S6 — Proteomic analysis of fusobacterial culture samples. [file mbio.03732-24-s0006.tif]

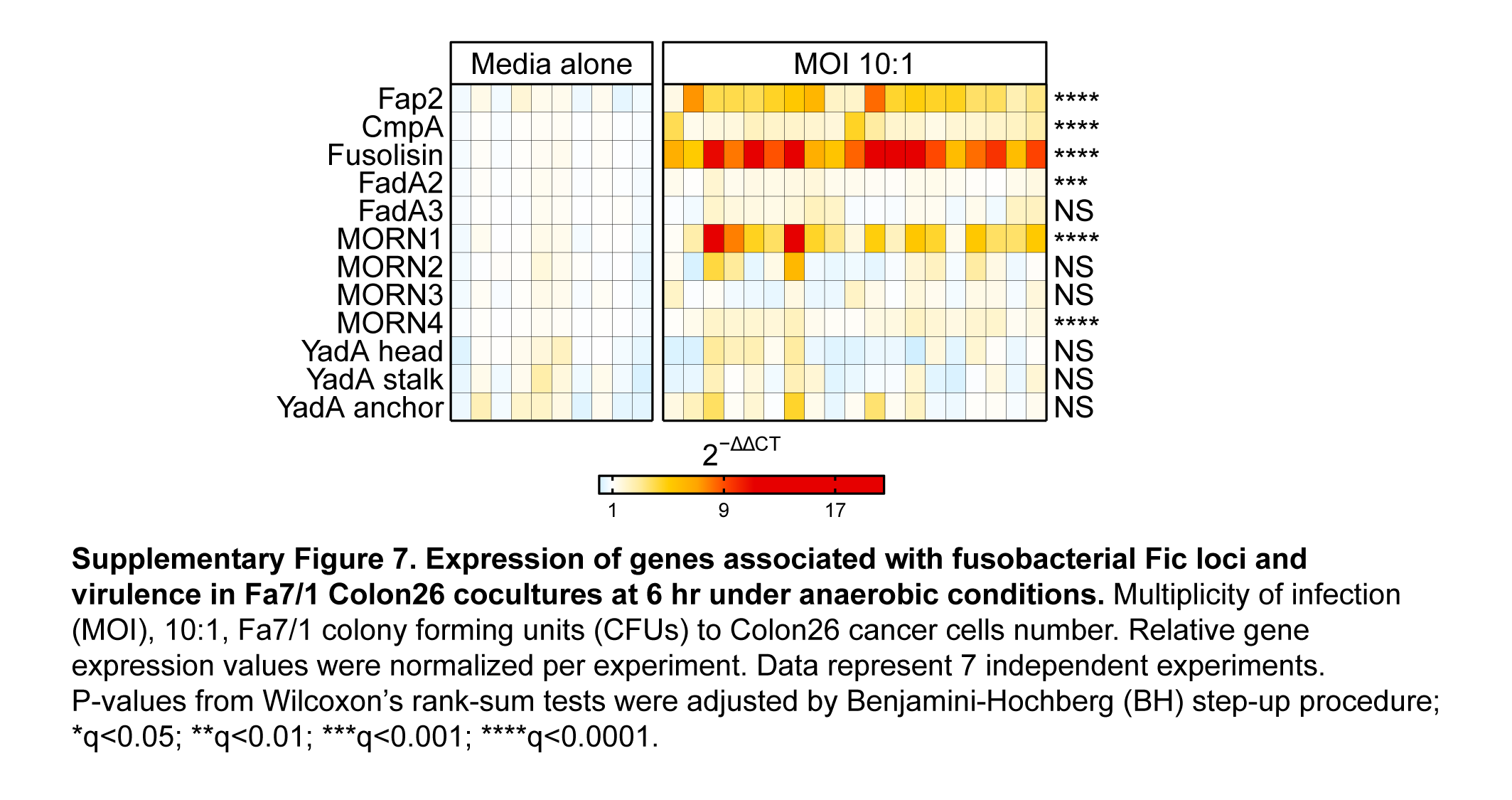

Supplement: Fig. S7 — Expression of genes associated with fusobacterial Fic loci and virulence in Fa7/1 Colon26 cocultures at 6 h under anaerobic conditions. [file mbio.03732-24-s0007.tif]
